# Supplementary material for: Predicting 1, 2 and 3 year emergent referable diabetic retinopathy and maculopathy using deep learning
Source: Commun Med (Lond). 2024 Aug 21;4:167. doi: 10.1038/s43856-024-00590-z (PMC11339445; doi:10.1038/s43856-024-00590-z)
Supplement: Supplementary file 2 — Supplementary Material [file 43856_2024_590_MOESM2_ESM.pdf]

# Supplementary Information

## Predicting 1, 2 and 3 year emergent referable diabetic retinopathy and maculopathy using deep learning

Paul Nderitu[1,2*]; Joan M. Nunez do Rio[2,3]; Laura Webster[4]; Samantha Mann[4]; M. Jorge Cardoso[3]; Marc Modat[3]; David Hopkins[5]; Christos Bergeles[3+]; Timothy L. Jackson[1,2+]

[1]Section of Ophthalmology, Faculty of Life Sciences and Medicine, King's College London, London, WC2R 2LS, UK.

[2]King's Ophthalmology Research Unit (KORU), Department of Ophthalmology, King's College Hospital, London, SE5 9RS, UK.

[3]School of Biomedical Engineering & Imaging Sciences, King's College London, London SE1 7EU, UK.

[4]Department of Ophthalmology, St Thomas' Hospital, London, SE1 7EH, UK.

[5]Institute of Diabetes, Endocrinology and Obesity, King's Health Partners, London, SE5 9RS, UK.

*Correspondence: p.nderitu@doctors.org.uk

[+]These authors jointly supervised this work

**TABLE OF CONTENTS**


Supplementary methods ..... 3
Study population and datasets ..... 3
Data curation ..... 3
Pretraining and longitudinal datasets ..... 3
Model development ..... 4
Risk factor characteristics data pre-processing ..... 4
Image data pre-processing ..... 4
Training augmentations ..... 4
Image and tabular DLS pretraining ..... 5
Image and tabular DLS longitudinal training ..... 5
Multimodal DLS ..... 6
Image ablation analysis ..... 6
Image DLS attribution analysis ..... 6
Tabular DLS attribution analysis ..... 6
Supplementary Table S1. Diabetic retinopathy screening grading definitions ..... 8
Supplementary Table S2. Summary of diabetic eye screening procedures ..... 9
Supplementary Figure S3. Deep learning system training schema for the 1 and 3 year prediction intervals ..... 10
Supplementary Table S4. Pretraining dataset and non-valid visits characteristics ..... 12
Supplementary Table S5. Training augmentations and hyperparameters ..... 13
Supplementary Table S6. Individual-level tabular, image and multimodal DLS performance ..... 14
Supplementary Table S7. Sensitivity and specificity for tabular, image and multimodal DLS ..... 15
Supplementary Figure S8A. Internal test False negative cases as a function of threshold ..... 16
Supplementary Figure S8B. External test False negative cases as a function of threshold ..... 17
Supplementary Figure S9A. Internal test Tabular, image and multimodal DLS positive and negative predictive value curves ..... 18
Supplementary Figure S9B. External test Tabular, image and multimodal DLS positive and negative predictive value curves ..... 19
Supplementary Figure S10. Internal test Mean image DLS attributions analysis for the 1 year prediction interval ..... 20
Supplementary Figure S11. External test Mean image DLS attributions analysis for the 1 year prediction interval ..... 21
Supplementary Figure S12. Internal test Mean image DLS attributions analysis for the 2 year prediction interval ..... 22
Supplementary Figure S13. External test Mean image DLS attributions analysis for the 1 year prediction interval ..... 23
Supplementary Figure S14. Internal test Mean image DLS attributions analysis for the 3 year prediction interval ..... 24
Supplementary Figure S15. External test Mean image DLS attributions analysis for the 3 year prediction interval ..... 25
Supplementary Figure S16. Single eye image DLS attributions for the 1 year prediction interval ..... 26
Supplementary Figure S17. Single eye image DLS attributions analysis for the 2 year prediction interval ..... 27
Supplementary Figure S18. Single eye image DLS attributions analysis for the 3 year prediction interval ..... 28
Supplementary Figure S19. Internal test Tabular DLS attributions analysis for the 1 year prediction interval ..... 29
Supplementary Figure S20. External test Tabular DLS attributions analysis for the 1 year prediction interval ..... 30
Supplementary Figure S21. Tabular DLS attributions analysis for the 2 year prediction interval ..... 31
Supplementary Figure S22. External test Tabular DLS attributions analysis for the 1 year prediction interval ..... 32
Supplementary Figure S23. Tabular DLS attributions for the 3 year prediction interval ..... 33
Supplementary Figure S24. External test Tabular DLS attributions analysis for the 1 year prediction interval ..... 34
References ..... 35
TRIPOD Checklist: Prediction model development and validation ..... 36

# SUPPLEMENTARY METHODS

## Study population and datasets

Diabetic eye screening programme (DESP) data from south-east London (SEL-DESP) were used for the development and internal test sets, whilst screening data from Birmingham, Solihull and Black Country (BSBC-DESP), a geographically independent UK diabetic retinopathy (DR) screening service, were used external testing. The UK DESP follows established national screening committee grading definitions[1] and protocols[2] which are summarised in supplementary table S1 and S2. All UK graders must undertake comprehensive training and are required to complete ~10 tests per year and need to attain an average sensitivity of >85% for referable DR detection on a varying test set.[3] Input data included colour fundal photographs (CFPs), and risk factor characteristics of age, sex, self-reported ethnicity, diabetes mellitus (DM) type, DM duration, visual acuity, and the index of multiple deprivation (IMD) rank. The IMD is a measure of relative deprivation based on UK postcodes, and is derived using 39 separate indicators across seven weighted domains.[4] The IMD is calculated for small neighbourhood areas which are ranked from 1 (least-deprived) to 32,844 (most deprived).[4] The UK DESP imaging protocol requires at least two 45° CFPs (*fovea and optic-disc centred field*) be taken after mydriasis.[5] Images from SEL-DESP and BSBC-DESP were captured using a wide range of approved cameras (supplementary table S2). DR and maculopathy grades, as documented by DESP graders, were extracted and used to derive the training and evaluation labels. The UK DESP uses multi-level DR grading whereby a primary grader identifies the presence or absence of DR, with the exceptions of proliferative DR cases which can be referred to the hospital eye service directly by the primary grader.[6] A secondary grader determines if DR is referable or non-referable in positive cases, and also reviews a random 10% sample of negative cases from primary grading as a quality assurance step to detect and quantify the rate of missed cases. A tertiary senior grader performs arbitration in cases of disagreement.

### *Data curation*

CFPs which were duplicates, missing the patient ID, corrupt, from an ineligible pathway (*slit-lamp biomicroscopy referral, non-DR referral or digital surveillance attendance*), or from ungradable eyes as defined by DESP graders were initially excluded. The curation of the remaining images was achieved using a sequence of deep learning systems (DLS) whose development and validation is described in detail in our prior work.[7] Briefly, curation DLS were trained to detect laterality, retinal presence, retinal field and gradability using a subset of ophthalmologist-labelled images from SEL-DESP. Curation DLS were used to select a pair of fovea-centred (*macula field*) and optic-disc-centred (*nasal field*) CFPs with the highest gradability prediction score from the pool of available gradable images per eye, with non-retinal, non-protocol field, single-field only, unidentifiable laterality and low gradability scoring images excluded (figure 1).[7] Images from eyes missing DR or maculopathy grades were also excluded at this stage. Curation DLS were applied to both the SEL-DESP and BSBC-DESP datasets. Additional details for the BSBC-DESP dataset including its composition and additional curation are published elsewhere.[8] Following data curation, 1,181,858 SEL-DESP and 66,286 BSBC-DESP CFPs were selected.

### *Pretraining and longitudinal datasets*

The median [interquartile range] number of visits in the development, internal and external test were 3 [2-4], 3 [2-4], and 3 [2-5] respectively, with a mean time ± standard deviation of 443 ± 139 days, 442 ± 138 days and 415 ± 156 days between any two visits respectively. Starting from the most recent attendance, the sequence of visits per eye were all searched to determine the first pair of visits 1, 2 or 3 years apart within a 2, 4 or 6 month tolerance respectively, termed the baseline and predict visit pair (figure 2, supplementary figure S5A and S5B). Eyes were excluded if no valid pairs could be found from the sequence of available visits. After applying this selection process, the mean time between visits for 1, 2 and 3 year interval cohorts in the development / internal test sets were 382 ± 21 days / 382 ± 21 days, 778 ± 43 days / 776 ± 43 days and 1,126 ± 110 days / 1,126 ± 110 days respectively with 370 ± 21 days, 743 ± 39 days and 1,110 ± 65 days for the external test set respectively. Eyes without a valid pair of visits at any of the aforementioned intervals in the development set were used for pretraining, whilst those in the internal and external test sets without valid longitudinal data were excluded (figure 1). Individuals without valid longitudinal data were younger, had marginally shorter DM duration, with a higher proportion being non-white or having type 1 DM compared to individuals with valid longitudinal data (supplementary table S4, table 1).

## Model development

The image DLS comprised of two EfficientNet-V2-s models[9] (one per CFP field, no weight sharing), concatenated at the final feature vector. EfficientNetV2 has state-of-the-art classification performance with rapid training, and 6.8x smaller model size.[9] Additionally, prior studies have demonstrated that EfficientNet models has better performance than a number of other models (VGG, Xception, InceptionV3, InceptionResNetV2, DenseNets, ResNets, NASNets) on the adjacent task of DR classification.[10,11] Our early experiments also showed that on the adjacent task of DR detection the use of 2 models independently for each image field was equal to using one model for both imaged fields (concatenated at the input) (referable DR/maculopathy detection AUROC 0.971 vs 0.972 for 2-field/1-model vs 2-field/2-models approaches). However the 2-field, 2-model approach was potentially advantageous because if there are independent prognostic features in each imaged field, but which differed between fields, each model could specialise to each field to learn these features specific to the given field. The tabular DLS were based on TabNet, a high-performance neural network for tabular data learning that can reportedly outperforms decision tree algorithms.[12] Early experiments also informed our tabular DLS choice. Namely we trained both a fully connected network and a non-DL based model (Adaboost) to predict incident referable DR/maculopathy within 2 years. Internal test results showed an ensemble of the image DLS with TabNet as the tabular DLS performed the best (AUROC 0.845) compared to an ensemble with a full connected network (AUROC 0.840) or Adabaoost (AUROC 0.826). DLS models were developed using opencv v4.5.5, albumentations v1.0.3, timm v0.4.12, torch v1.10.1, torchmetrics v0.6.2, and pytorch-lightning v1.5.8 python libraries, with DLS attributions computed using captum v0.4.1. Other python libraries used for data analyses included numpy v1.21.5, pandas v1.3.5, scikit-learn v1.0.2, matplotlib v3.5.1, scipy v1.7.3 and statsmodels v0.13.2. A Windows 10 Pro workstation with a 32 core Intel Xenon CPU, 128GB of RAM and x2 24GB Quadra RTX P6000 GPUs was used for DLS development and internal testing. External testing was performed on a cloud-based trusted research environment with a GPU with 12GB of RAM.

### *Risk factor characteristics data pre-processing*

Missing continuous risk factor characteristics data (age, DM duration, IMD rank) were imputed using the training set median values based on the baseline visit for the respective longitudinal interval dataset or from all visits for the non-longitudinal pretraining dataset. Minimal imputation was required as <2% of continuous data variables were missing from any dataset partition. Continuous data were standardised using training set derived *mean* and *standard deviation* values (*equation 1*). Categorical variables were passed through an embedding layer with a '*Not Specified*' category used to represent missing categorical variables.

$$(1)\quad \frac{x - mean}{standard\ deviation}$$

### *Image data pre-processing*

Images were cropped to the fundus circle mask using the minimum image dimension (SEL-DESP) or using a mask of the grayscale image filtered to keep pixel values greater than a tolerance value of 7 (BSBC-DESP). Cropped images were then resized to 512x512 using bilinear interpolation. The image size was informed by prior observations by Krause *et al* that DR detection performance gains are marginal with image sizes >450px.[13] Left eye images were flipped horizontally to a right eye orientation to reduce inter-image variance and aid image DLS training as convolutional-filters are not rotationally invariant. Images were normalised to [0, 1] range then standardised per channel using ImageNet *mean* = [0.485, 0.456, 0.406] and *standard deviation* = [0.229, 0.224, 0.225] constants (*equation 1*, figure 2, supplementary figure S5A and 5B).

### *Training augmentations*

Random augmentations were independently applied per CFP field during image DLS training with a probability of 0.5. Training augmentations included brightness (+/- 0.15), contrast (-0.25, +0.5), hue shift (+/- 7), saturation shift (-63, +127) and vertical flip. Additionally, gaussian blur (+/- 5) was applied with a probability of 0.1 to mimic the occurrence of sub-optimally focused images (supplementary table S3).

## *Image and tabular DLS pretraining*

Image and tabular DLS were pretrained to classify DR (R0: no DR, R1: mild-moderate DR, R2: moderate-severe DR and R3A: proliferative DR) and maculopathy (M0: no referable maculopathy and M1: referable maculopathy) using the pretraining dataset which was divided 88% for training and 12% for tuning. Aforementioned augmentations were also applied during image DLS pretraining. RMSProp with momentum 0.9 were used to minimise the proportional class weighted mean categorical cross entropy loss for DR classification and the positive class weighted binary cross entropy loss for maculopathy classification (*equation 2*). The binary entropy loss component, $Loss_{mac}$, was multiplied by the loss weighting parameter $\alpha = 0.25$, a value ascertained from bayesian hyperparameter search (HyperOpt algorithm) of possible values [0.1, 0.25, 0.5, 0.75, 1.0] after 10 pretraining trials for a maximum of 10 epochs each. Image DLS batch size was 12 whilst tabular DLS batch size was 64. The starting learning rate was 0.0001, held constant for 10 epochs and reduced by 1/10 at epoch 10 and again by 1/100 from epoch 30. Other hyperparameters included dropout (0.2) and pretraining weight regularisation parameter ($\lambda_{pre}$=0.0001). Hyperparameters were the same for the image and tabular DLS with additional tabular DLS hyperparameters of attention layer (64), prediction layer (16) attention steps (3) and attention update (γ, 1.3) (supplementary table S3). Image and tabular DLS were trained for a maximum of 60 epochs with an early stopping criterion of a 3 epoch plateau in the mean area-under-the receiver operating characteristic (AUROC) for DR classification on the tuning set. The best performing DLS was selected using the highest mean AUROC for DR classification on the tuning set. Interval vs external non-longitudinal test set AUROC for the pretraining DR classification task were 0.926 vs 0.931 for R0, 0.889 vs 0.870 for R1, 0.976 vs 0.969 for R2, 0.972 vs 0.962 for R3A, and 0.967 vs 0.963 for M1 in the SEL-DESP and BSBC-DESP datasets respectively.

(2)

$$Loss_{pre} = Loss_{ret} + \alpha * Loss_{mac} + \lambda_{pre}\|\theta\|_2$$

$$Loss_{ret} = -\frac{1}{N}\sum\sum_{c}^{C}[w_{Rc}R_c\, log(\widehat{R}_c)]$$

$$Loss_{mac} = -\frac{1}{N}\sum\left[w_{M+}Mlog(\widehat{M}) + (1-M)log(1-\widehat{M})\right]$$

*where N = No. of samples, $C$ = No. retinopathy classes, $w_{Rc}$ = Proportional retinopathy class weight, $R_c$ = Retinopathy ground truth for the class, $\widehat{R_c}$ = Retinopathy prediction for the class, $w_{M+}$ = Positive maculopathy class weight, M = Maculopathy ground truth, $\widehat{M}$ = Maculopathy prediction, α = Maculopathy loss weight parameter, $\lambda_{pre}$ = Pretraining regularization parameter, $\theta$ = Model parameters.*

## *Image and tabular DLS longitudinal training*

Image and tabular DLS were trained to concurrently predict emergent referable DR, referable maculopathy or either as a multilabel binary objective using the development longitudinal dataset which was divided 88% for training and 12% for tuning. Detecting mild-moderate DR (R1) at the baseline visit was an additional auxiliary binary objective (*equation 3*, figure 2, supplementary figure S5A and 5B). The auxiliary binary objective of detecting mild-moderate DR at baseline was chosen as this is one of the most significant risk factors for progression to referable DR.[14,15] Therefore, training the DLS to detect mild-moderate DR at baseline was hypothesised to significantly improve its performance in predicting referable DR as the learnt features from cross-sectional disease detection would likely be prognostic. Indeed the auxiliary task of baseline DR detection improved performance on the primary task of predicting progression to referable DR for all intervals, with internal test 2-year AUROC for $R2^+$ | M1 for the tabular DLS improving from 0.70 to 0.72, and the image DLS AUROC improving from 0.75 to 0.80. Pretraining improved DLS performance in the subsequent progression prediction task for all intervals, with 2-year AUROC for $R2^+$ | M1 for the tabular DLS improving from 0.70 to 0.74, and the image DLS AUROC improving from 0.81 to 0.82. AdamW optimiser was used to minimise the positive class weighted mean binary cross entropy loss for referable progression forecasting (multilabel) and the positive class weighted binary cross entropy for baseline DR detection (auxiliary), with 1:1 weighting between losses. Image DLS batch size was 12 whilst tabular DLS batch size was 64. The starting learning rate was 0.0001, held constant for 10 epochs and reduced by 1/10 at epoch 10 and again by 1/100 from epoch 30. Other hyperparameters included dropout (0.2) and the longitudinal weight regularisation parameter ($\lambda_{long}$=0.0001). Hyperparameters were the same for the image and tabular DLS with additional tabular DLS hyperparameters of attention layer (64), prediction layer (16) attention steps (3) and attention update (γ, 1.3) (supplementary table S3). Image and tabular DLS were trained for a maximum of 60 epochs with an early stopping criterion of a 3 epoch plateau in the mean tuning AUROC for the primary task. The best performing DLS was selected using the highest tuning mean AUROC for the primary multilabel binary task.

(3) $$Loss_{long} = Loss_{prog} + Loss_{aux} + \lambda_{long}\|\theta\|_2$$

$$Loss_{prog} = -\frac{1}{N}\sum\sum_{c}^{C}\left[w_{P_c+}P_c\log\left(\hat{P}_c\right) + (1 - P_c)\log\left(1 - \hat{P}_c\right)\right]$$

$$Loss_{aux} = -\frac{1}{N}\sum\left[w_{B+}Blog\left(\hat{B}\right) + (1 - B)log\left(1 - \hat{B}\right)\right]$$

*where N = No. of samples, C = No. progression classes,* $w_{P_c+}$ *= Positive progression class weight, P = Progression ground truth,* $\hat{P}$ *= Progression prediction,* $w_{B+}$ *= Positive baseline (auxiliary) class weight, B = Baseline (auxiliary) ground truth,* $\hat{B}$ *= Baseline (auxiliary) prediction,* $\lambda_{long}$ *= Longitudinal regularization parameter,* $\theta$ *= Model parameters.*

### *Multimodal DLS*

The multimodal DLS was defined by taking the mean of the image and tabular DLS predictions (prediction-level fusion) at inference. Experiments showed that prediction-level fusion performed better than end-to-end training with late fusion defined as the concatenation of the final feature vector of the image and tabular DLS. The 2-year internal test set multimodal DLS AUROC for prediction-level fusion vs late fusion end-to-end training were 0.92 (95% CI: 0.87-0.96) vs 0.88 (0.82-0.94), 0.84 (0.82-0.87) vs 0.83 (0.81-0.86) and 0.85 (0.82-0.87) vs 0.83 (0.81-0.86) for predicting emergent referable DR, maculopathy or either respectively. On the basis of this findings, prediction-level fusion was used for all multimodal DLS.

## Image ablation analysis

To determine regions within CFPs that are important to the trained image DLS predictions, we applied a circular mask, whose diameter was defined as a percentage of the image width at test-time and progressively revealed or removed CFP areas of the macula or nasal field. Starting with an unmasked nasal field and fully-masked macula field, we progressively unmasked the macula field from the centre to periphery until both fields were visible (figure 4). We then progressively masked the nasal field from the periphery to the centre until only the macula image was visible. We evaluated the test set AUROC at each stage of masking/unmasking to determine the importance of the visible macula and nasal field area to the trained image DLS performance (the DLS were not retrained) with the ablation analysis performed for 1, 2 and 3 year intervals per outcome.

## Image DLS attribution analysis

To further delineate image regions associated with image DLS predictions, we computed attribution maps using GradCAM[16] for 40 (internal test set) and 19 (external test set) randomly drawn positive eyes (as per the ground truth) for 1, 2 and 3 year intervals relative to each outcome. Because left eye images are flipped to a right eye orientation during preprocessing, macula and nasal fields between cases are approximately aligned. Therefore, computing the average attribution between samples (*with assumed alignment*) results in a heatmap of the CFP image regions which are most associated with the respective outcome predictions for positive cases (supplementary figures S13-15). Additionally, we computed the eye level attributions GradCAM[16] for 3 example eyes from the internal test set with respect to the referable DR and maculopathy DLS output to allow for a better localisation of potential lesions which are pertinent to image DLS predictions (supplementary figures S16-18).

## Tabular DLS attribution analysis

Tabular DLS attribution analyses were performed for 1, 2 and 3 year intervals with respect to each outcome. Using 1,000 randomly drawn test set cases (*positive and negative*), we used integrated gradients[17] to evaluate the overall change in tabular DLS predictions (*increase, decrease, or unchanged*) as we interpolated from a reference to test risk factor

characteristic value. For categorical variables, interpolation was performed in the embeddings space and the attributions vector summed to scalar value. The reference point was a white, 60y old female with type II diabetes of 0y duration, with 6/6 (0.0 logMAR) vision, and a median level of deprivation (IMD rank 16,422). The reference was chosen to reflect a low-risk case. An increase or decrease in the tabular DLS prediction relative to this reference suggests that the tabular DLS ascribed a higher or lower risk of progression relative to the reference ‘low risk’ factor characteristic value. The change in the prediction, reflecting the effect the characteristic had on the tabular DLS predictions, were plotted for all 1,000 samples with each risk factor characteristic coloured according to its original value from blue to red for low to high values for continuous variables or a fixed colour for categorical variables (supplementary figures S19-21).

## SUPPLEMENTARY TABLE S1. DIABETIC RETINOPATHY SCREENING GRADING DEFINITIONS

| Grade | Description | Criteria |
|---|---|---|
| Retinopathy | | |
| R0 | No DR | • No retinal DR lesions |
| R1 | Mild to moderate non-proliferative DR | • Presence of microaneurysms or retinal haemorrhages<br>• Exudates or cotton wool spots in the presence of DR features |
| R2* | Moderate to severe non-proliferative DR | • Presence of multiple blot haemorrhages, venous beading or intraretinal microvascular abnormalities |
| R3A* | Proliferative DR | • New features of proliferative disease, namely, new vessels at the disk or elsewhere, preretinal/vitreous haemorrhage, new preretinal fibrosis or new tractional retinal detachment<br>• Previous treatment for proliferative DR that has not been deemed stable by the treating ophthalmologist<br>• New features indicating reactivation of proliferation, or potentially sight threatening change from fibrous proliferation with respect to previously reference images |
| R3S | Stable-treated proliferative DR | • Evidence of peripheral retinal laser treatment and stable retina with respect to reference images taken at or shortly after discharge from the hospital eye service. |
| Maculopathy | | |
| M0 | No referable diabetic maculopathy | • Referable diabetic maculopathy criteria not met |
| M1* | Referable diabetic maculopathy | • Exudate ≤1 disc diameter from the fovea<br>• ≥1/2 a disc area of exudates within the macula<br>• Microaneurysm ≤1 disc diameter from the fovea AND visual acuity ≤6/12 |

DR=Diabetic retinopathy. *Referable DR or referable maculopathy as per UK national screening committee definitions[1].

## SUPPLEMENTARY TABLE S2. SUMMARY OF DIABETIC EYE SCREENING PROCEDURES

| Variable | South-East London Diabetic Eye Screening Programme | Birmingham, Solihull and Black Country Diabetic Eye Screening Programme |
|---|---|---|
| Country [City] | United Kingdom [London] | United Kingdom [Birmingham] |
| Setting [Sites] | Diabetic Eye Screening Programme<br>[27 Hospital and Community Sites] | Diabetic Eye Screening Programme<br>[110 Hospital and Community Sites] |
| Screening Pathway | Routine digital screening | Routine digital screening |
| Study Duration | Sept 2013 to Dec 2019 | Sept 2013 to Dec 2019 |
| Eligibility | 12 years and older<br>DM diagnosis<br>Light perception visual acuity or better in at least one eye | 12 years and older<br>DM diagnosis<br>Light perception visual acuity or better in at least one eye |
| Routine Screening Interval | Annual | Annual |
| Mydriasis | Yes | Yes |
| Retinal Cameras | Majority of images: Topcon [NW6, NW8]<br><br>Minority of images: Canon CR2, Topcon Triton OCT Fundal Camera | Majority of images: Topcon [NW6]<br><br>Minority of images: CR-DGi, Nidek AFC-210, Kowa Alpha 8 |
| Image Type | Colour photographs | Colour photographs |
| Image Format | jpg (84%), nef (16%) | jpg (100%) |
| Imaging Protocol | Two-Field<br>45° fovea-centred (macula) & optic-disc-centred (nasal) | Two-Field<br>45° fovea-centred (macula) & optic-disc-centred (nasal) |
| Native Image Resolutions | [1,960 x 1,934] to [6,000 x 4,000] | [1,152 x 1,728] to [6,000 x 4,000] |
| Grading System | $1^0$ Grader: No DR/DR<br>$2^0$ Grader: Non-referable/Referable DR, 10% of No DR cases Regraded.<br>$3^0$ Grader: Arbitration | $1^0$ Grader: No DR/DR<br>$2^0$ Grader: Non-referable/Referable DR, 10% of No DR cases Regraded.<br>$3^0$ Grader: Arbitration |
| Grading Platform Recommendations | Desktop monitor with minimum 1,080 pixels vertical resolution | Desktop monitor with minimum 1,080 pixels vertical resolution |
| Quality Assurance & Audit Processes | Local and national audits, key performance indicators, grader qualifications, grader training and testing (~10/year), intergrader agreement reports, 10% no DR regrading to define missed cases, local quality assurance inspections, national guidelines, policies and service specifications. | Local and national audits, key performance indicators, grader qualifications, grader training and testing (~10/year), intergrader agreement reports, 10% no DR regrading to define missed cases, local quality assurance inspections, national guidelines, policies and service specifications. |

DM=Diabetes mellitus. DR=Diabetic retinopathy.

## SUPPLEMENTARY FIGURE S3. DEEP LEARNING SYSTEM TRAINING SCHEMA FOR THE 1 AND 3 YEAR PREDICTION INTERVALS

### A. 1 Year Prediction Interval

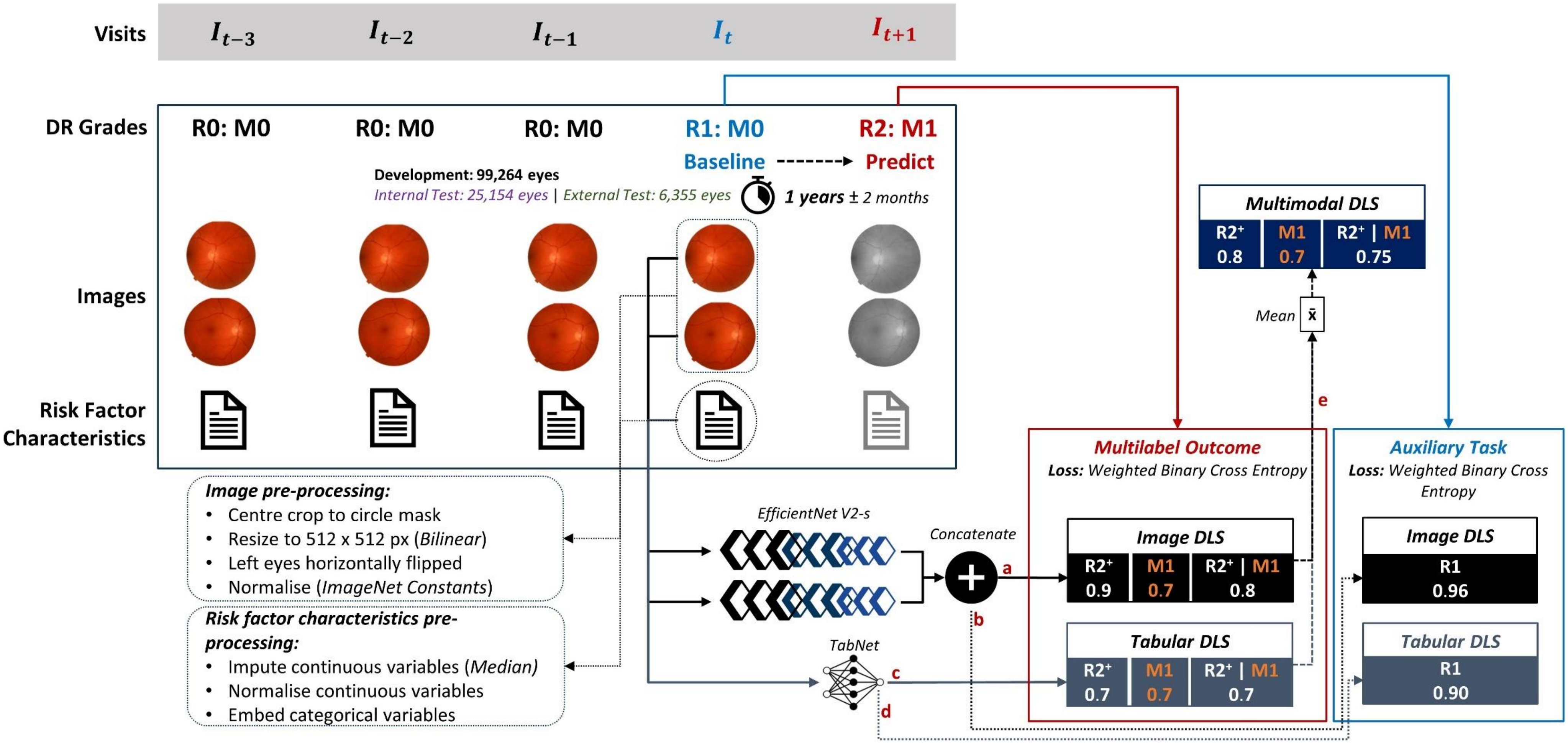


Data pre-processing and training schema used to develop DLS for the 1-year prediction interval. Two-field images from the baseline visit (blue) are used as input to the image DLS, whilst risk factor characteristics are inputs for the tabular DLS. Baseline and predict visits (red) must be 1 year from the baseline visit ±2 months. DLS predict if emergent referable disease occurs between the baseline and predict visit. Image and tabular DLS are trained to predict the binary progression outcomes (a & c) with the ground truth from the predict visit (red continuous line), with an auxiliary task of detecting baseline DR (b & d) with the ground truth from the baseline visit (blue continuous line). Numbers below each outcome indicative of the prediction score. The multimodal DLS is formed by taking a mean of the tabular and image DLS predictions at test time (e). DLS=Deep learning system. $I_t$=Visit number in the sequence. R0=No DR. R1=Mild-moderate DR. R2=Moderate-severe DR. M0=No referable maculopathy. M1=Referable maculopathy. $R2^+$ | M1=Referable DR or maculopathy. $R2^+$=Referable DR.

## B. 3 Year Prediction Interval

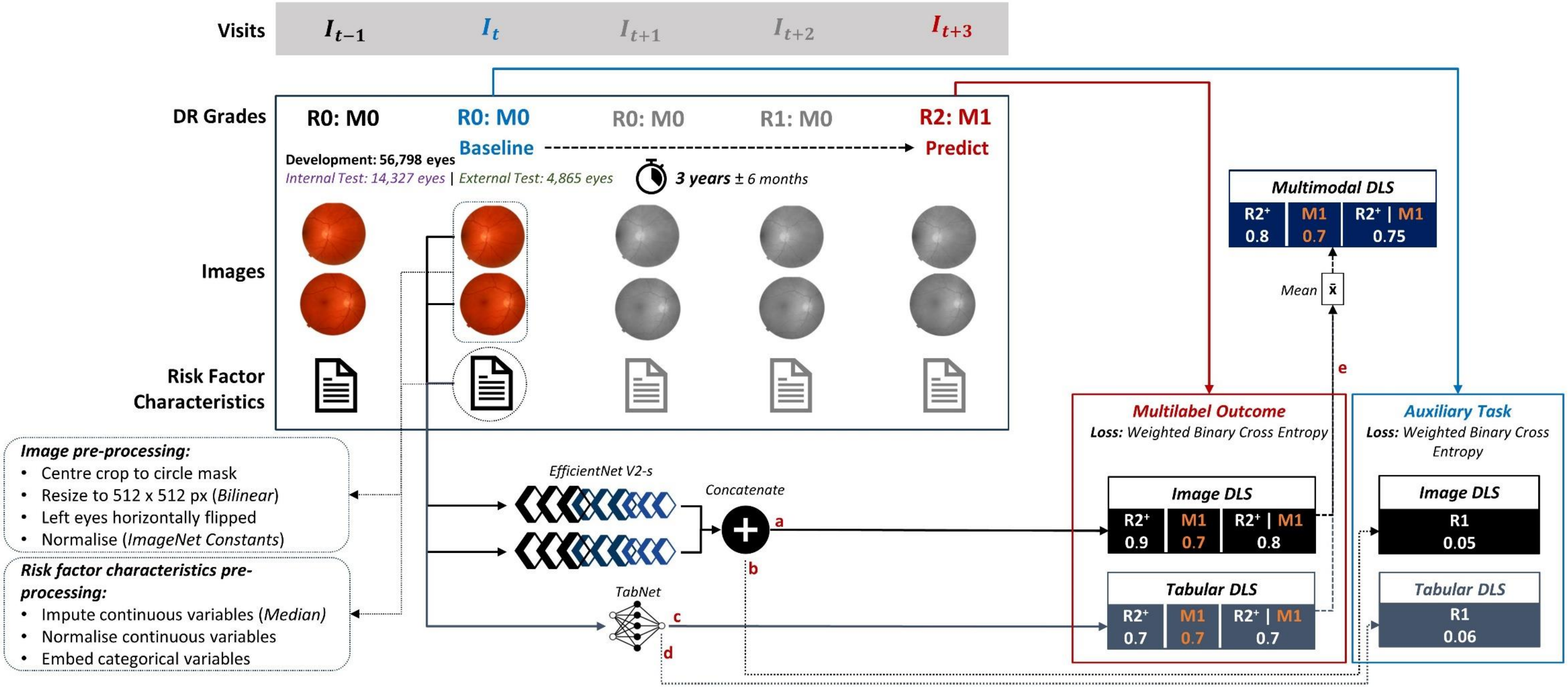


Data pre-processing and training schema used to develop DLS for the 3-year prediction interval. Two-field images from the baseline visit (blue) are used as input to the image DLS, whilst risk factor characteristics are inputs for the tabular DLS. Baseline and predict visits (red) must be 3 years from the baseline visit ±6 months. DLS predict if emergent referable disease occurs between the baseline and predict visit. Image and tabular DLS are trained to predict the binary progression outcomes (a & c) with the ground truth from the predict visit (red continuous line), with an auxiliary task of detecting baseline DR (b & d) with the ground truth from the baseline visit (blue continuous line). Numbers below each outcome indicative of the prediction score. The multimodal DLS is formed by taking a mean of the tabular and image DLS predictions at test time (e). DLS=Deep learning system. $I_t$=Visit number in the sequence. R0=No DR. R1=Mild-moderate DR. R2=Moderate-severe DR. M0=No referable maculopathy. M1=Referable maculopathy. $R2^+$ | M1=Referable DR or maculopathy. $R2^+$=Referable DR.

## SUPPLEMENTARY TABLE S4. PRETRAINING DATASET AND NON-VALID VISITS CHARACTERISTICS

| Characteristic | | Pretraining Dataset: (Development Set - Included) Unique eyes = 51,502 Individuals = 26,346 | | No Valid Longitudinal Visits (Internal Test Set - Excluded) Unique eyes = 12,593 Individuals = 6,443 | | No Valid Longitudinal Visits (External Test Set - Excluded) Unique eyes = 2,435 Individuals = 1,282 | |
|---|---|---|---|---|---|---|---|
| | | N or [mean] | % or (SD) | N or [mean] | % or (SD) | N or [mean] | % or (SD) |
| Age | Years | [58] | (16) | [59] | (16) | [60] | (16) |
| Sex | Male | 28,119 | 54.6 | 7,007 | 55.6 | 1,310 | 53.8 |
| | Female | 23,354 | 45.3 | 5,578 | 44.3 | 1,051 | 43.2 |
| | Not Specified | 29 | 0.1 | 8 | 0.1 | 74 | 3.0 |
| Ethnicity | White | 24,910 | 48.4 | 6,102 | 48.5 | 1,194 | 49.0 |
| | Black | 14,035 | 27.3 | 3,381 | 26.9 | 164 | 6.7 |
| | South Asian | 3,425 | 6.7 | 820 | 6.5 | 621 | 25.5 |
| | Other Asian | 3,174 | 6.2 | 835 | 6.6 | 50 | 2.1 |
| | Mixed | 1,506 | 2.9 | 385 | 3.1 | 40 | 1.6 |
| | Other | 1,427 | 2.7 | 356 | 2.8 | 41 | 1.7 |
| | Not Specified | 3,025 | 5.9 | 714 | 5.7 | 74 | 3.0 |
| Visual Acuity | LogMAR | [0.18] | (0.31) | [0.17] | (0.31) | [0.16] | (0.36) |
| IMD | Rank | [13,398] | (7,844) | [13,332] | (7,845) | [8,756] | (8,243) |
| DM Type | Type 2 | 46,440 | 90.2 | 11,309 | 89.8 | 1,152 | 47.3 |
| | Type 1 | 4,284 | 8.3 | 1,097 | 8.7 | 112 | 4.6 |
| | Other | 96 | 0.2 | 36 | 0.3 | 5 | 0.2 |
| | Not Specified | 682 | 1.3 | 151 | 1.2 | 1,166 | 47.9 |
| DM Duration | Years | [7.1] | (8.2) | [7.0] | (8.4) | [3.5] | (3.9) |
| DR Grades | R0 | 36,768 | 71.4 | 12,593 | 72.6 | 1,722 | 70.7 |
| | R1 | 11,373 | 22.1 | 9,145 | 21.1 | 527 | 21.6 |
| | R2 | 2,305 | 4.5 | 536 | 4.3 | 103 | 4.2 |
| | R3A | 780 | 1.5 | 195 | 1.6 | 62 | 2.6 |
| | R3S[1] | 276 | 0.5 | 55 | 0.4 | 21 | 0.9 |
| Maculopathy Grades | M0 | 45,838 | 89.0 | 11,257 | 89.4 | 2,134 | 87.6 |
| | M1 | 5,664 | 11.0 | 1,336 | 10.6 | 301 | 12.4 |

[1]Not used for deep learning system pretraining. Characteristics computed using the baseline (first) visit in eyes with multiple visits. SD=Standard deviation. LogMAR=Logarithm of the minimal angle of resolution. IMD=Index of multiple deprivation. DM=Diabetes mellitus. DR=Diabetic retinopathy. N=Unique Eyes.

## SUPPLEMENTARY TABLE S5. TRAINING AUGMENTATIONS AND HYPERPARAMETERS

| Descriptor | Pretraining | | Longitudinal training | |
|---|---|---|---|---|
| | Image DLS | Tabular DLS | Image DLS | Tabular DLS |
| Network | EfficientNet-V2-s[9] (x2) | TabNet[12] | EfficientNet-V2-s[9] (x2) | TabNet[12] |
| Resolution | 512 x 512 pixels per field | - | 512 x 512 pixels per field | - |
| Batch Size | 12 | 64 | 12 | 64 |
| Augmentations | Vertical flip: p=0.5<br>Brightness ±0.15: p=0.5<br>Contrast -0.25, +0.5: p=0.5<br>Hue shift ±7: p=0.5<br>Saturation shift -63, +127: p=0.5<br>Gaussian blur ±5: p=0.1 | - | Vertical flip: p=0.5<br>Brightness ±0.15: p=0.5<br>Contrast -0.25, +0.5: p=0.5<br>Hue shift ±7: p=0.5<br>Saturation shift -63, +127: p=0.5<br>Gaussian blur ±5: p=0.1 | - |
| Other Hyperparameters | - | Attention layer: 64<br>Attention steps: 3<br>Attention update ($\gamma$): 1.3<br>Prediction layer: 16 | - | Attention layer: 64<br>Attention steps: 3<br>Attention update ($\gamma$): 1.3<br>Prediction layer: 16 |
| Dropout<br>Last layer | 0.2 | 0.2 | 0.2 | 0.2 |
| Initialisation | ImageNet | Random | Pretrained image DLS | Pretrained tabular DLS |
| Losses | Categorical + 0.25 * binary cross entropy loss | Categorical + 0.25 * binary cross entropy loss | Multilabel binary + auxiliary binary cross entropy loss<br>(all weighted x1) | Multilabel binary + auxiliary binary cross entropy loss<br>(all weighted x1) |
| Optimisation & Regularisation | RMSProp (Momentum 0.9)<br>Learning rate: 0.0001<br>$L_2$ Weight regularisation: 0.0001 | RMSProp (Momentum 0.9)<br>Learning rate: 0.0001<br>$L_2$ Weight regularisation: 0.0001 | AdamW<br>Learning rate: 0.0001<br>$L_2$ Weight regularisation: 0.0001 | AdamW<br>Learning rate: 0.0001<br>$L_2$ Weight regularisation: 0.0001 |
| Learning rate Schedule & Max Epochs | x1/10 at 10 epochs then x1/100 at 30 epochs for max 60 epochs | x1/10 at 10 epochs then x1/100 at 30 epochs for max 60 epochs | x1/10 at 10 epochs then x1/100 at 30 epochs for max 60 epochs | x1/10 at 10 epochs then x1/100 at 30 epochs for max 60 epochs |
| Early Stopping | 3 epoch tuning AUROC plateau | 3 epoch tuning AUROC plateau | 3 epoch tuning AUROC plateau | 3 epoch tuning AUROC plateau |
| Checkpoint | Max mean tuning AUROC | Max mean tuning AUROC | Max mean tuning AUROC | Max mean tuning AUROC |

DLS=Deep learning system. AUROC=Area-under-the receiver operating characteristic.

## SUPPLEMENTARY TABLE S6. INDIVIDUAL-LEVEL TABULAR, IMAGE AND MULTIMODAL DLS PERFORMANCE

| Model | Input Data | Outcome | Internal Test AUROC (95%CI) Year 1 Individuals 12,800 | Internal Test AUROC (95%CI) Year 2 Individuals 9,228 | Internal Test AUROC (95%CI) Year 3 Individuals 7,345 | External Test AUROC (95%CI) Year 1 Individuals 3,264 | External Test AUROC (95%CI) Year 2 Individuals 2,967 | External Test AUROC (95%CI) Year 3 Individuals 2,519 |
|---|---|---|---|---|---|---|---|---|
| Tabular DLS TabNet | Risk Factor Characteristics[1] | $R2^+$ \| M1 | 0.71 (0.68-0.73) | 0.73 (0.70-0.76) | 0.70 (0.67-0.73) | 0.77 (0.62-0.71) | 0.69 (0.64-0.75) | 0.64 (0.58-0.69) |
| | | $R2^+$ | 0.82 (0.76-0.88) | 0.77 (0.69-0.86) | 0.72 (0.64-0.80) | 0.74 (0.61-0.86) | 0.80 (0.61-0.89) | 0.69 (0.56-0.81) |
| | | M1 | 0.70 (0.67-0.73) | 0.73 (0.70-0.76) | 0.70 (0.67-0.73) | 0.67 (0.62-0.71) | 0.68 (0.62-0.74) | 0.64 (0.59-0.70) |
| Image DLS EfficientNet-V2-s [X2] | Two-Field Colour Fundal Photographs | $R2^+$ \| M1 | 0.85 (0.83-0.87) | 0.82 (0.79-0.85) | 0.76 (0.73-0.79) | 0.87 (0.83-0.91) | 0.77 (0.70-0.83) | 0.77 (0.71-0.82) |
| | | p vs tabular DLS | <0.001 | <0.001 | 0.004 | <0.001 | 0.028 | 0.001 |
| | | $R2^+$ | 0.95 (0.92-0.98) | 0.87 (0.80-0.94) | 0.84 (0.78-0.89) | 0.95 (0.92-0.99) | 0.92 (0.86-0.98) | 0.89 (0.77-1.00) |
| | | p vs tabular DLS | <0.001 | 0.071 | 0.017 | 0.002 | 0.049 | 0.017 |
| | | M1 | 0.84 (0.82-0.86) | 0.82 (0.79-0.85) | 0.76 (0.73-0.79) | 0.86 (0.82-0.90) | 0.75 (0.68-0.82) | 0.76 (0.70-0.82) |
| | | p vs tabular DLS | <0.001 | <0.001 | 0.005 | <0.001 | 0.065 | 0.002 |
| Multimodal DLS EfficientNet-V2-s [X2] & TabNet | Two-Field Colour Fundal Photographs & Risk Factor Characteristics[1] | $R2^+$ \| M1 | 0.85 (0.83-0.87) | 0.84 (0.81-0.86) | 0.78 (0.75-0.81) | 0.86 (0.82-0.90) | 0.78 (0.73-0.84) | 0.78 (0.73-0.83) |
| | | p vs tabular DLS | <0.001 | <0.001 | <0.001 | <0.001 | <0.001 | <0.001 |
| | | p vs image DLS | 0.896 | 0.029 | 0.004 | 0.355 | 0.146 | 0.384 |
| | | $R2^+$ | 0.94 (0.91-0.98) | 0.89 (0.84-0.95) | 0.86 (0.80-0.91) | 0.95 (0.93-0.97) | 0.93 (0.88-0.98) | 0.92 (0.83-1.00) |
| | | p vs tabular DLS | <0.001 | 0.002 | <0.001 | <0.001 | 0.006 | 0.001 |
| | | p vs image DLS | 0.334 | 0.412 | 0.395 | 0.904 | 0.297 | 0.435 |
| | | M1 | 0.84 (0.82-0.86) | 0.84 (0.81-0.86) | 0.78 (0.75-0.81) | 0.85 (0.81-0.90) | 0.77 (0.71-0.83) | 0.77 (0.72-0.83) |
| | | p vs tabular DLS | <0.001 | <0.001 | <0.001 | <0.001 | 0.002 | <0.001 |
| | | p vs image DLS | 0.893 | 0.034 | 0.009 | 0.295 | 0.195 | 0.404 |

[1]Risk factor characteristics=Age, sex, ethnicity, diabetes type, diabetes duration, best visual acuity, index of multiple deprivation rank. AUROC=Area-under-the receiver operating characteristic. CI=Confidence interval. DLS=Deep learning system. $R2^+$ | M1=Referable DR or maculopathy. $R2^+$=Referable DR. M1=Referable maculopathy. Significant at $p<0.017$, Bonferroni adjusted for the three predefine outcomes. Confidence intervals (95%) and significance were calculated using DeLong's method.[18]

## SUPPLEMENTARY TABLE S7. SENSITIVITY AND SPECIFICITY FOR TABULAR, IMAGE AND MULTIMODAL DLS

| Model | Input Data | Outcome | Confusion Matrix | Internal Test | | | | | | External Test | | | | | |
|---|---|---|---|---|---|---|---|---|---|---|---|---|---|---|---|
| | | | | Year 1 Eyes: 25,154 | | Year 2 Eyes: 18,100 | | Year 3 Eyes: 14,327 | | Year 1 Eyes: 6,355 | | Year 2 Eyes: 5,506 | | Year 3 Eyes: 4,865 | |
| Tabular DLS TabNet | Risk Factor Characteristics[1] | $R2^+$ \| M1 | TP \| FP | 382 | 12,245 | 265 | 8,784 | 262 | 7,912 | 114 | 3,355 | 86 | 2,919 | 94 | 2,838 |
| | | | FN \| TN | 95 | 12,432 | 66 | 8,985 | 65 | 6,088 | 29 | 2,857 | 21 | 2,480 | 24 | 2,337 |
| | | | Sensitivity \| Specificity | 0.80 | 0.50 | 0.80 | 0.51 | 0.80 | 0.43 | 0.80 | 0.46 | 0.80 | 0.46 | 0.80 | 0.40 |
| | | $R2^+$ | TP \| FP | 56 | 6,402 | 34 | 8,115 | 50 | 8,856 | 15 | 2,850 | 17 | 1,756 | 17 | 2,507 |
| | | | FN \| TN | 14 | 18,682 | 9 | 9,942 | 12 | 5,409 | 4 | 3,756 | 4 | 3,729 | 4 | 2,337 |
| | | | Sensitivity \| Specificity | 0.80 | 0.74 | 0.80 | 0.55 | 0.80 | 0.38 | 0.79 | 0.59 | 0.81 | 0.68 | 0.81 | 0.48 |
| | | M1 | TP \| FP | 350 | 12,685 | 246 | 8,722 | 236 | 7,840 | 104 | 3,205 | 76 | 3,034 | 89 | 2,761 |
| | | | FN \| TN | 87 | 12,032 | 62 | 9,070 | 60 | 6,191 | 26 | 3,020 | 19 | 2,377 | 21 | 1,994 |
| | | | Sensitivity \| Specificity | 0.80 | 0.49 | 0.80 | 0.51 | 0.80 | 0.44 | 0.80 | 0.49 | 0.80 | 0.44 | 0.80 | 0.42 |
| Image DLS EfficientNet-V2-s [X2] | Two-Field Colour Fundal Photographs | $R2^+$ \| M1 | TP \| FP | 382 | 6,458 | 265 | 6,041 | 262 | 6,353 | 114 | 1,529 | 86 | 2,315 | 94 | 1,963 |
| | | | FN \| TN | 95 | 18,219 | 66 | 11,728 | 65 | 7,465 | 29 | 4,683 | 21 | 3,084 | 24 | 2,784 |
| | | | Sensitivity \| Specificity | 0.80 | 0.74 | 0.80 | 0.66 | 0.80 | 0.53 | 0.80 | 0.75 | 0.80 | 0.57 | 0.80 | 0.59 |
| | | $R2^+$ | TP \| FP | 56 | 1,015 | 34 | 2,319 | 50 | 4,713 | 15 | 856 | 17 | 928 | 17 | 801 |
| | | | FN \| TN | 14 | 24,069 | 9 | 15,738 | 12 | 9,552 | 4 | 5,480 | 4 | 4,557 | 4 | 4,043 |
| | | | Sensitivity \| Specificity | 0.80 | 0.96 | 0.80 | 0.87 | 0.80 | 0.67 | 0.79 | 0.86 | 0.81 | 0.83 | 0.81 | 0.83 |
| | | M1 | TP \| FP | 350 | 7,211 | 246 | 5,973 | 237 | 6,630 | 104 | 1,720 | 76 | 2,413 | 88 | 1,945 |
| | | | FN \| TN | 87 | 17,506 | 62 | 11,819 | 59 | 7,401 | 26 | 4,505 | 19 | 2,998 | 22 | 2,810 |
| | | | Sensitivity \| Specificity | 0.80 | 0.71 | 0.80 | 0.66 | 0.80 | 0.53 | 0.80 | 0.72 | 0.80 | 0.55 | 0.80 | 0.59 |
| Multimodal DLS EfficientNet-V2-s [X2] & TabNet | Two-Field Colour Fundal Photographs & Risk Factor Characteristics[1] | $R2^+$ \| M1 | TP \| FP | 382 | 6,321 | 265 | 5,275 | 262 | 5,835 | 114 | 1,214 | 86 | 2,154 | 94 | 1,967 |
| | | | FN \| TN | 95 | 18,356 | 66 | 12,494 | 65 | 8,165 | 29 | 4,998 | 21 | 3,245 | 24 | 2,780 |
| | | | Sensitivity \| Specificity | 0.80 | 0.74 | 0.80 | 0.70 | 0.80 | 0.58 | 0.80 | 0.80 | 0.80 | 0.60 | 0.80 | 0.59 |
| | | $R2^+$ | TP \| FP | 56 | 952 | 34 | 1,404 | 50 | 2,997 | 15 | 480 | 17 | 405 | 17 | 614 |
| | | | FN \| TN | 14 | 24,132 | 9 | 16,653 | 12 | 11,268 | 4 | 5,856 | 4 | 5,080 | 4 | 4,430 |
| | | | Sensitivity \| Specificity | 0.80 | 0.96 | 0.80 | 0.92 | 0.80 | 0.79 | 0.79 | 0.92 | 0.81 | 0.93 | 0.81 | 0.87 |
| | | M1 | TP \| FP | 350 | 7,621 | 246 | 5,463 | 237 | 5,895 | 104 | 1,464 | 76 | 2,290 | 88 | 1,942 |
| | | | FN \| TN | 87 | 17,096 | 62 | 12,329 | 59 | 8,136 | 26 | 4,761 | 19 | 3,121 | 22 | 2,813 |
| | | | Sensitivity \| Specificity | 0.80 | 0.69 | 0.80 | 0.69 | 0.80 | 0.58 | 0.80 | 0.76 | 0.80 | 0.58 | 0.80 | 0.59 |

[1]Risk factor characteristics=Age, sex, ethnicity, diabetes type, diabetes duration, best visual acuity, index of multiple deprivation rank. TP=True positive. FP=False positive. FN=False negative. TN=True negative. $R2^+$ | M1=Referable DR or maculopathy. $R2^+$=Referable DR. M1=Referable maculopathy.

## SUPPLEMENTARY FIGURE S8A. INTERNAL TEST FALSE NEGATIVE CASES AS A FUNCTION OF THRESHOLD

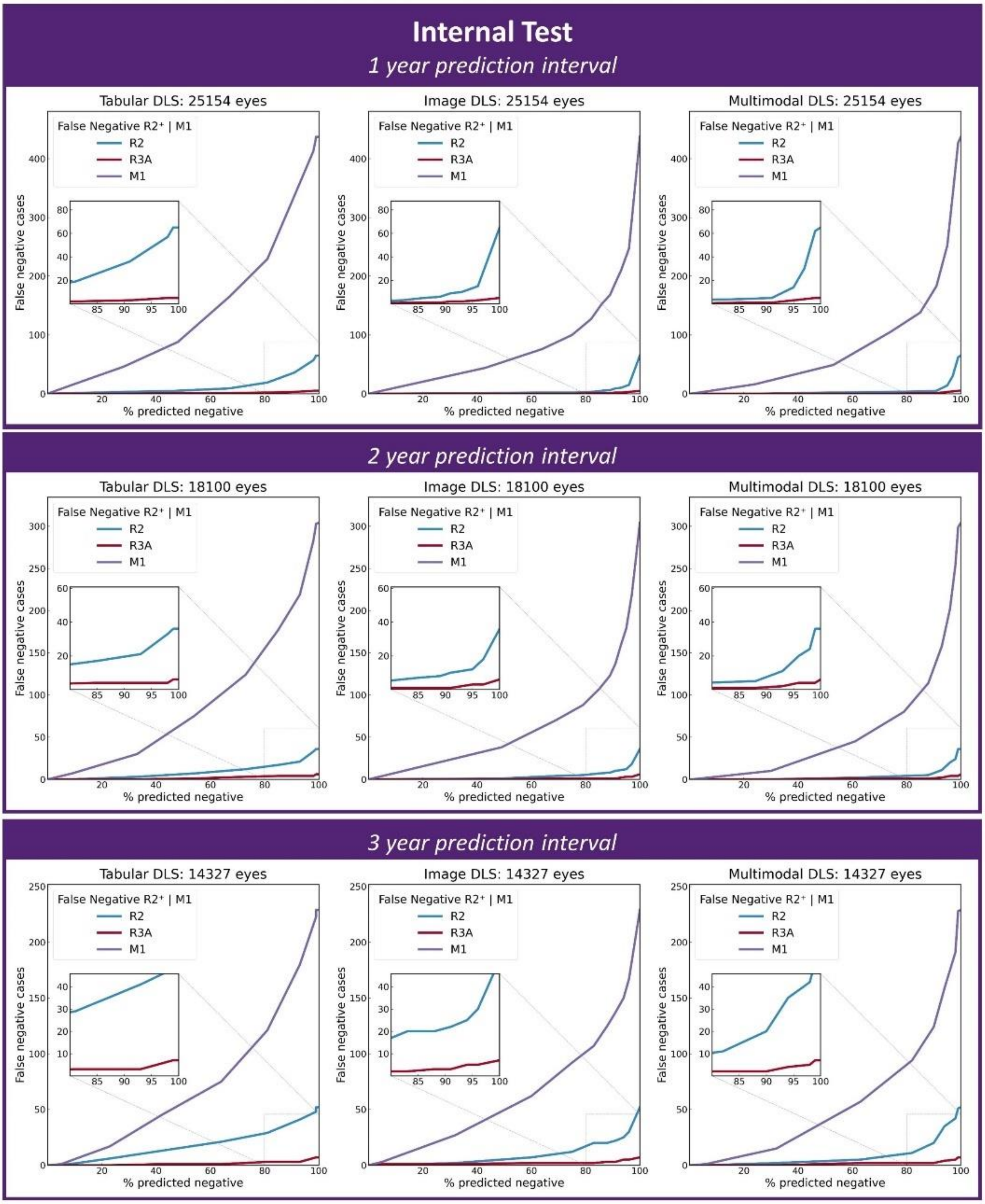


False negative cases as a function of threshold where the lowest threshold results in 0% of individuals being predicted as negative and the highest threshold results in 100% of individuals being predicted as negative for disease progression. Inset shows missed R2 and R3A cases at higher thresholds (≥80% predicted negative). The total eyes in the test set per interval are shown in the subplot title. DLS=Deep learning system. R2=Moderate-severe DR. R3A=Proliferative DR, M1=Referable maculopathy. $R2^+$ | M1=Referable DR or maculopathy.

## SUPPLEMENTARY FIGURE S8B. EXTERNAL TEST FALSE NEGATIVE CASES AS A FUNCTION OF THRESHOLD

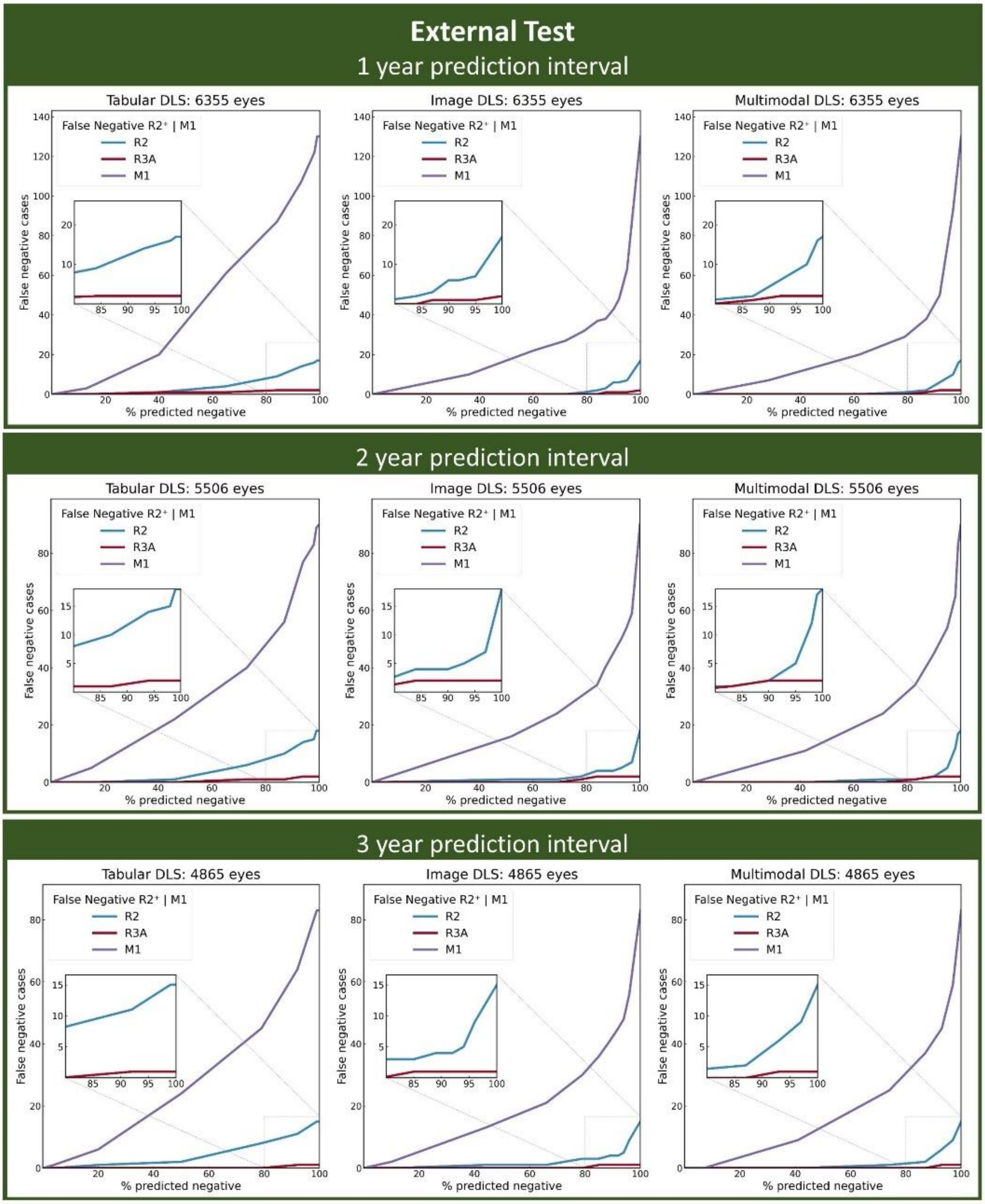


False negative cases as a function of threshold where the lowest threshold results in 0% of individuals being predicted as negative and the highest threshold results in 100% of individuals being predicted as negative for disease progression. Inset shows missed R2 and R3A cases at higher thresholds (≥80% predicted negative). The total eyes in the test set per interval are shown in the subplot title. DLS=Deep learning system. R2=Moderate-severe DR. R3A=Proliferative DR, M1=Referable maculopathy. $R2^+$ | M1=Referable DR or maculopathy.

## SUPPLEMENTARY FIGURE S9A. INTERNAL TEST TABULAR, IMAGE AND MULTIMODAL DLS POSITIVE AND NEGATIVE PREDICTIVE VALUE CURVES

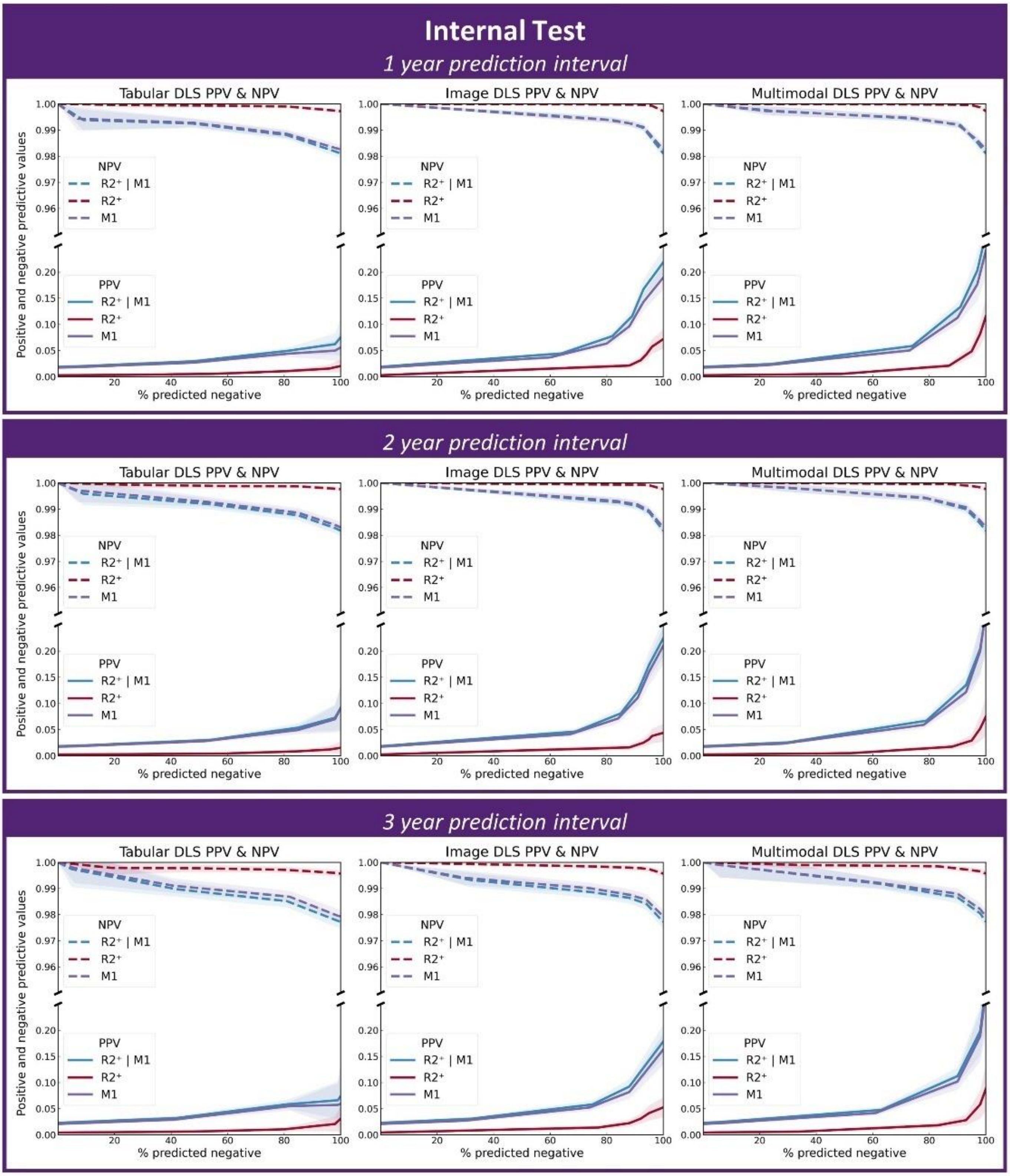


Positive and negative predictive values as a function of threshold where the lowest threshold results in 0% of individuals being predicted as negative and the highest threshold results in 100% of individuals being predicted as negative for disease progression. Shaded regions indicate 95% confidence intervals obtained using the Clopper-Pearson method (β distribution).[19] The y-axis is split to accommodate for the differences between positive and negative predictive values. PPV=Positive predictive value. NPV=Negative predictive value. DLS=Deep learning system. $R2^+$ | M1=Referable DR or maculopathy. $R2^+$=Referable DR. M1=Referable maculopathy.

## SUPPLEMENTARY FIGURE S9B. EXTERNAL TEST TABULAR, IMAGE AND MULTIMODAL DLS POSITIVE AND NEGATIVE PREDICTIVE VALUE CURVES

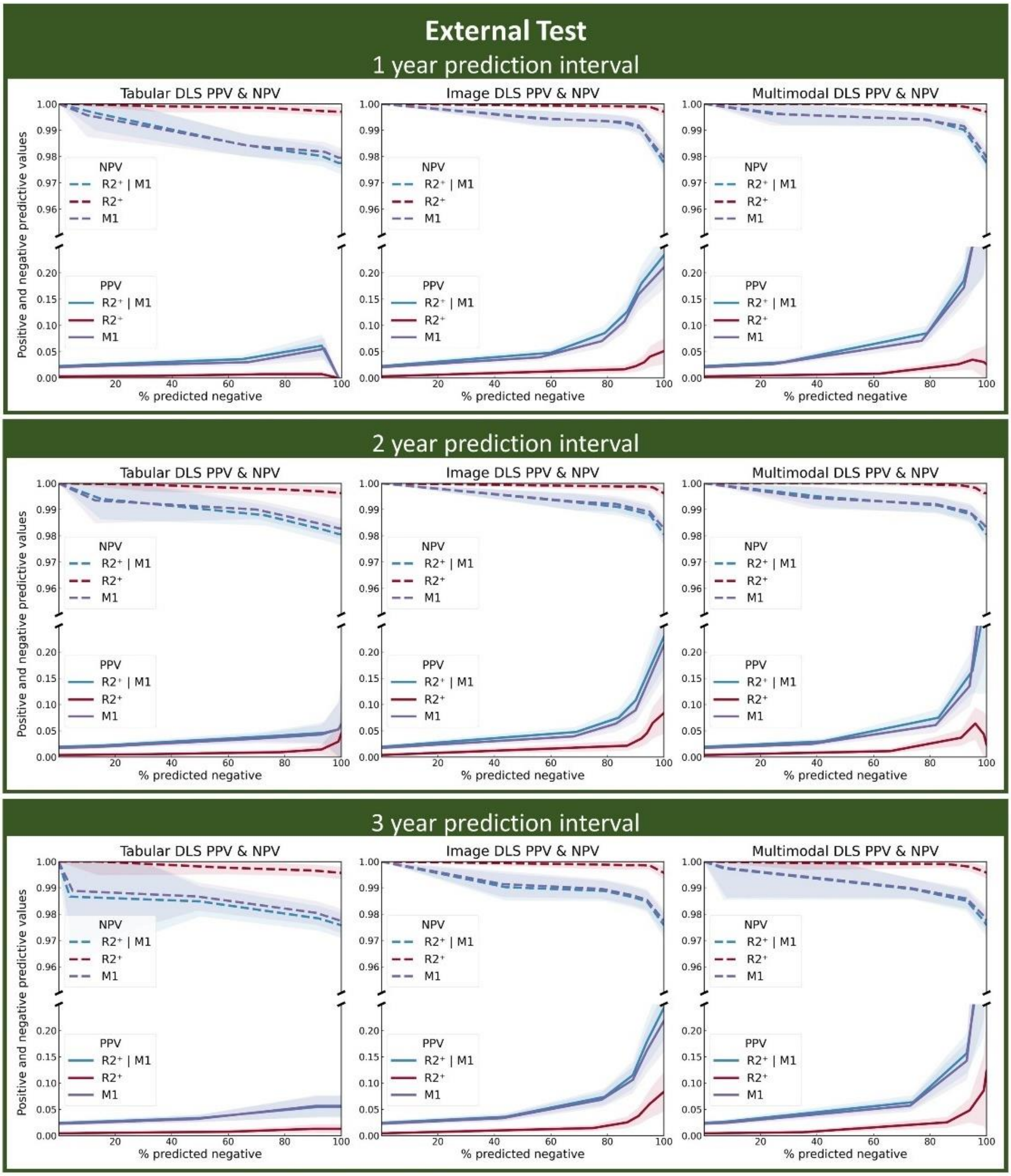


Positive and negative predictive values as a function of threshold where the lowest threshold results in 0% of individuals being predicted as negative and the highest threshold results in 100% of individuals being predicted as negative for disease progression. Shaded regions indicate 95% confidence intervals obtained using the Clopper-Pearson method (β distribution).[19] The y-axis is split to accommodate for the differences between positive and negative predictive values. PPV=Positive predictive value. NPV=Negative predictive value. DLS=Deep learning system. $R2^+$ | M1=Referable DR or maculopathy. $R2^+$=Referable DR. M1=Referable maculopathy.

## SUPPLEMENTARY FIGURE S10. INTERNAL TEST MEAN IMAGE DLS ATTRIBUTIONS ANALYSIS FOR THE 1 YEAR PREDICTION INTERVAL

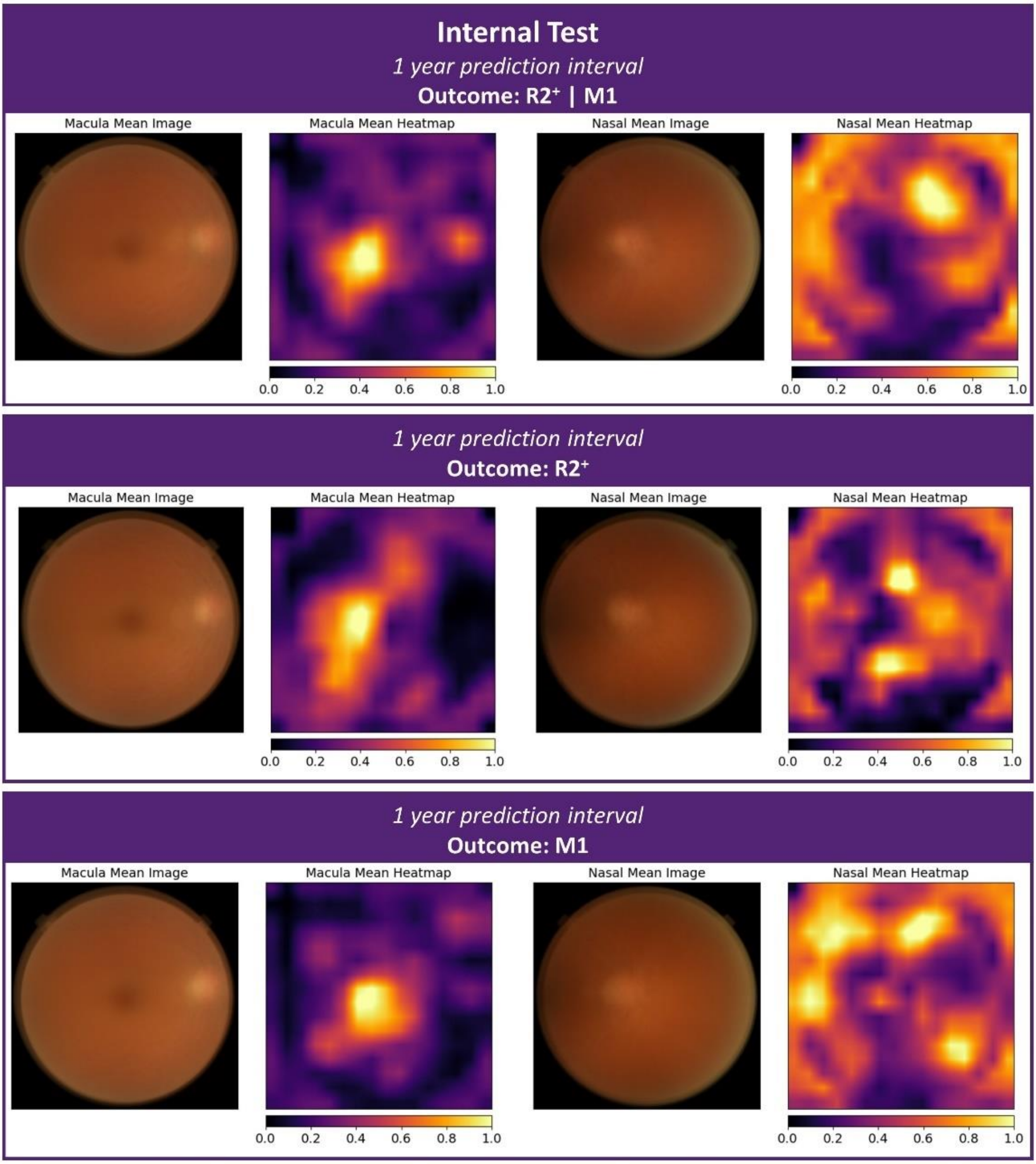


Mean heatmap computed using GradCAM[16] attribution method from 40 (SEL-DESP) randomly sampled positive cases (*based on the ground truth*) for the 1-year prediction interval. Left macula and nasal fields are horizontally flipped to right eye orientation during pre-processing. Computation of mean images assumes approximate alignment on the basis of this pre-processing which is supported by fact that the fovea and optic disc remain distinguishable in the mean image. $R2^+$ | M1=Referable DR or maculopathy. $R2^+$=Referable DR. M1=Referable maculopathy.

## SUPPLEMENTARY FIGURE S11. EXTERNAL TEST MEAN IMAGE DLS ATTRIBUTIONS ANALYSIS FOR THE 1 YEAR PREDICTION INTERVAL

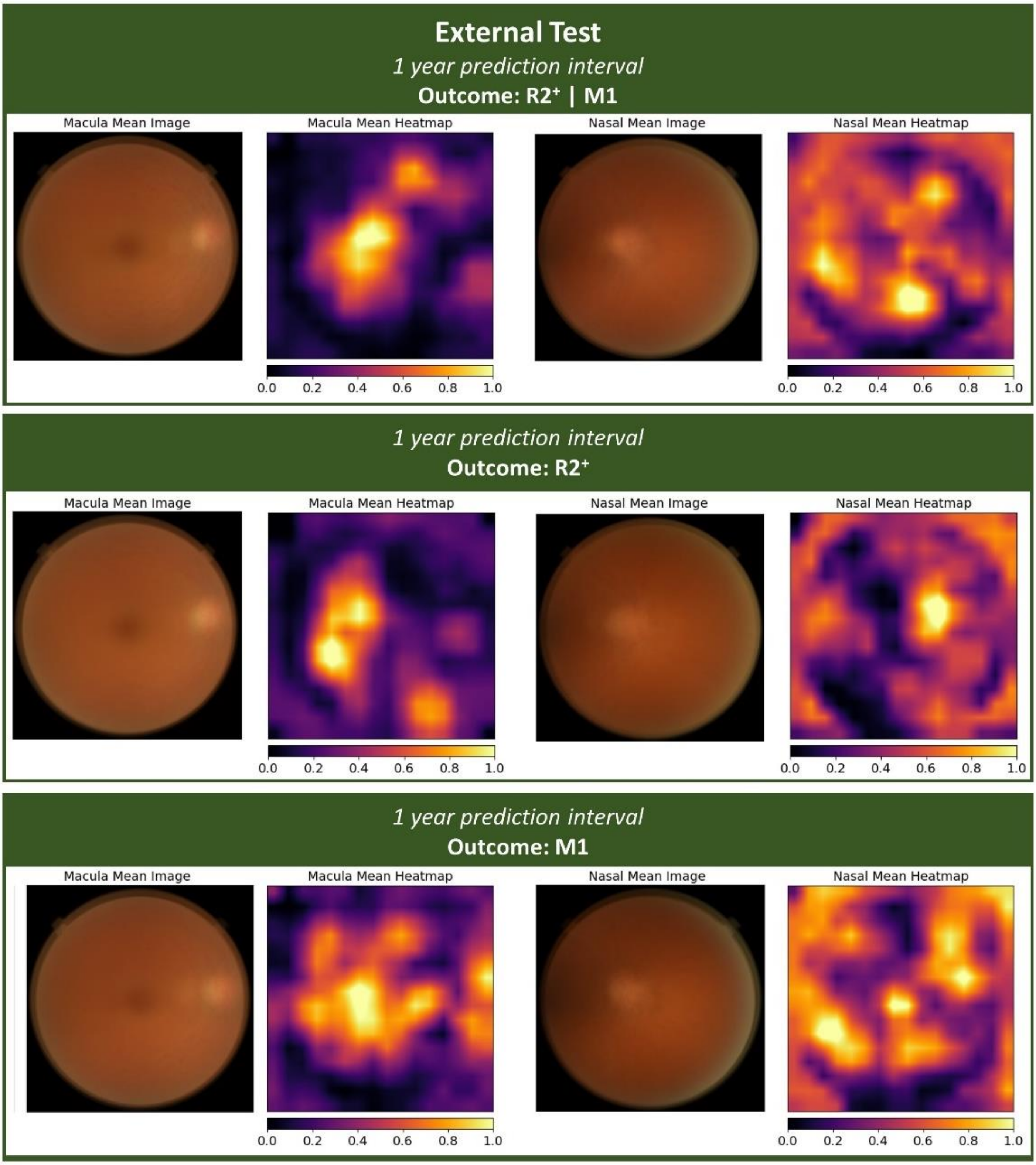


Mean heatmap computed using GradCAM[16] attribution method from 19 (BSBC-DESP) randomly sampled positive cases (*based on the ground truth*) for the 1-year prediction interval. Left macula and nasal fields are horizontally flipped to right eye orientation during pre-processing. Computation of mean images assumes approximate alignment on the basis of this pre-processing which is supported by fact that the fovea and optic disc remain distinguishable in the mean image. $R2^+$ | M1=Referable DR or maculopathy. $R2^+$=Referable DR. M1=Referable maculopathy.

## SUPPLEMENTARY FIGURE S12. INTERNAL TEST MEAN IMAGE DLS ATTRIBUTIONS ANALYSIS FOR THE 2 YEAR PREDICTION INTERVAL

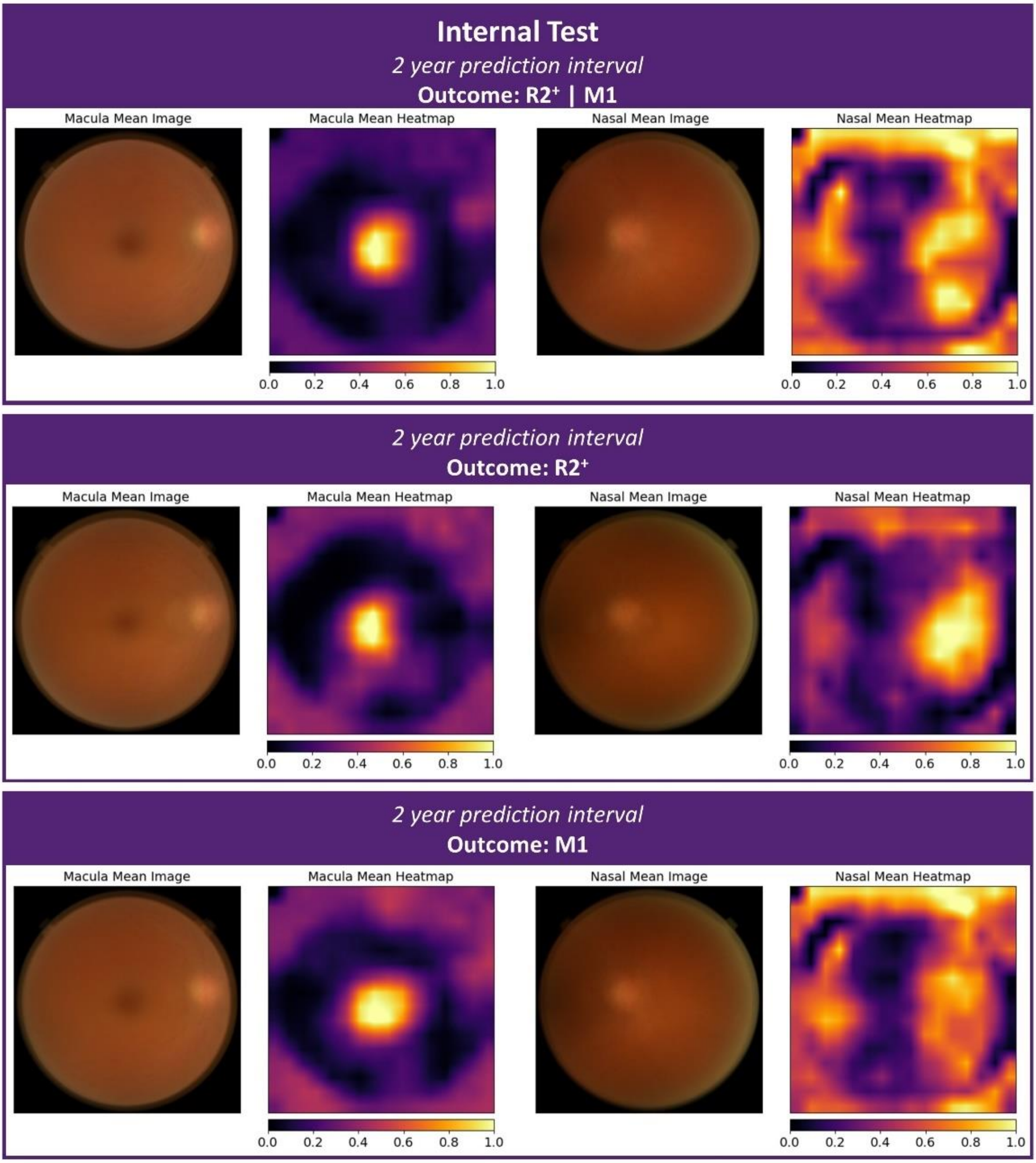


Mean heatmap computed using GradCAM[16] attribution method from 40 (SEL-DESP) randomly sampled positive cases (*based on the ground truth*) for the 2-year prediction interval. Left macula and nasal fields are horizontally flipped to right eye orientation during pre-processing. Computation of mean images assumes approximate alignment on the basis of this pre-processing which is supported by fact that the fovea and optic disc remain distinguishable in the mean image. $R2^+$ | M1=Referable DR or maculopathy. $R2^+$=Referable DR. M1=Referable maculopathy.

## SUPPLEMENTARY FIGURE S13. EXTERNAL TEST MEAN IMAGE DLS ATTRIBUTIONS ANALYSIS FOR THE 1 YEAR PREDICTION INTERVAL

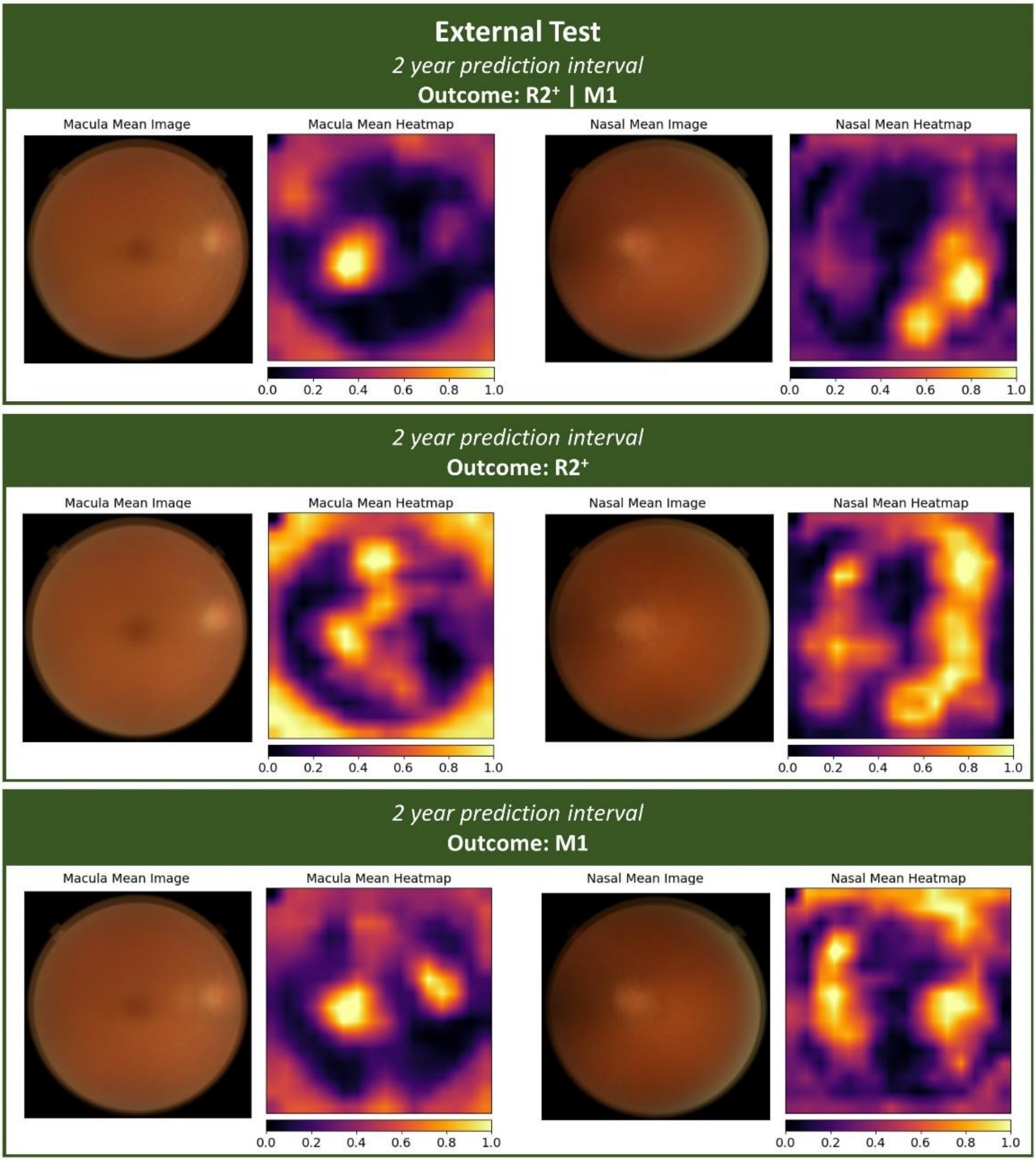


Mean heatmap computed using GradCAM[16] attribution method from 19 (BSBC-DESP) randomly sampled positive cases (*based on the ground truth*) for the 2-year prediction interval. Left macula and nasal fields are horizontally flipped to right eye orientation during pre-processing. Computation of mean images assumes approximate alignment on the basis of this pre-processing which is supported by fact that the fovea and optic disc remain distinguishable in the mean image. $R2^+$ | M1=Referable DR or maculopathy. $R2^+$=Referable DR. M1=Referable maculopathy.

## SUPPLEMENTARY FIGURE S14. INTERNAL TEST MEAN IMAGE DLS ATTRIBUTIONS ANALYSIS FOR THE 3 YEAR PREDICTION INTERVAL

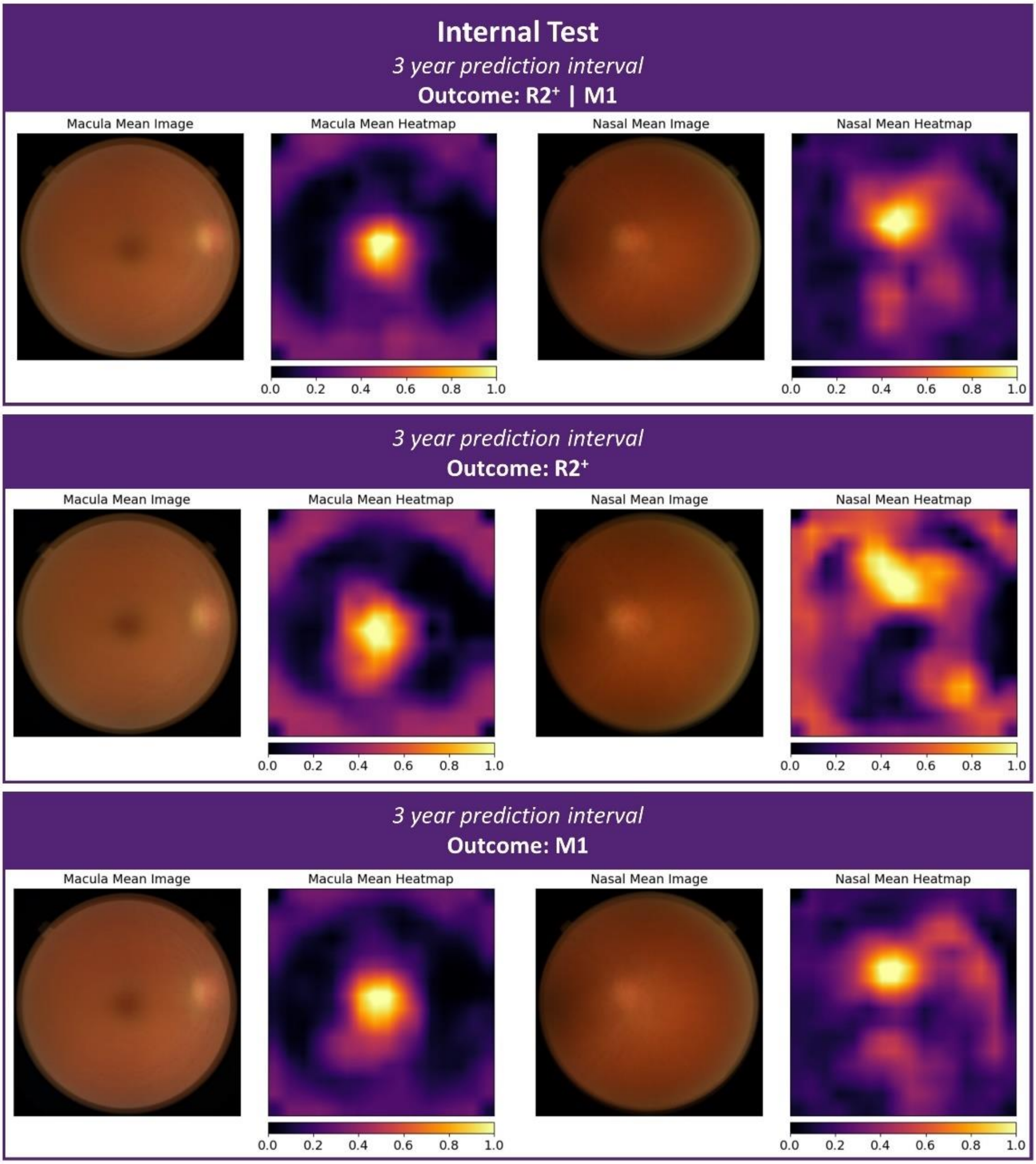


Mean heatmap computed using GradCAM[16] attribution method from 40 (SEL-DESP) randomly sampled positive cases (*based on the ground truth*) for the 3-year prediction interval. Left macula and nasal fields are horizontally flipped to right eye orientation during pre-processing. Computation of mean images assumes approximate alignment on the basis of this pre-processing which is supported by fact that the fovea and optic disc remain distinguishable in the mean image. $R2^+$ | M1=Referable DR or maculopathy. $R2^+$=Referable DR. M1=Referable maculopathy.

**SUPPLEMENTARY FIGURE S15. EXTERNAL TEST MEAN IMAGE DLS ATTRIBUTIONS ANALYSIS FOR THE 3 YEAR PREDICTION INTERVAL**

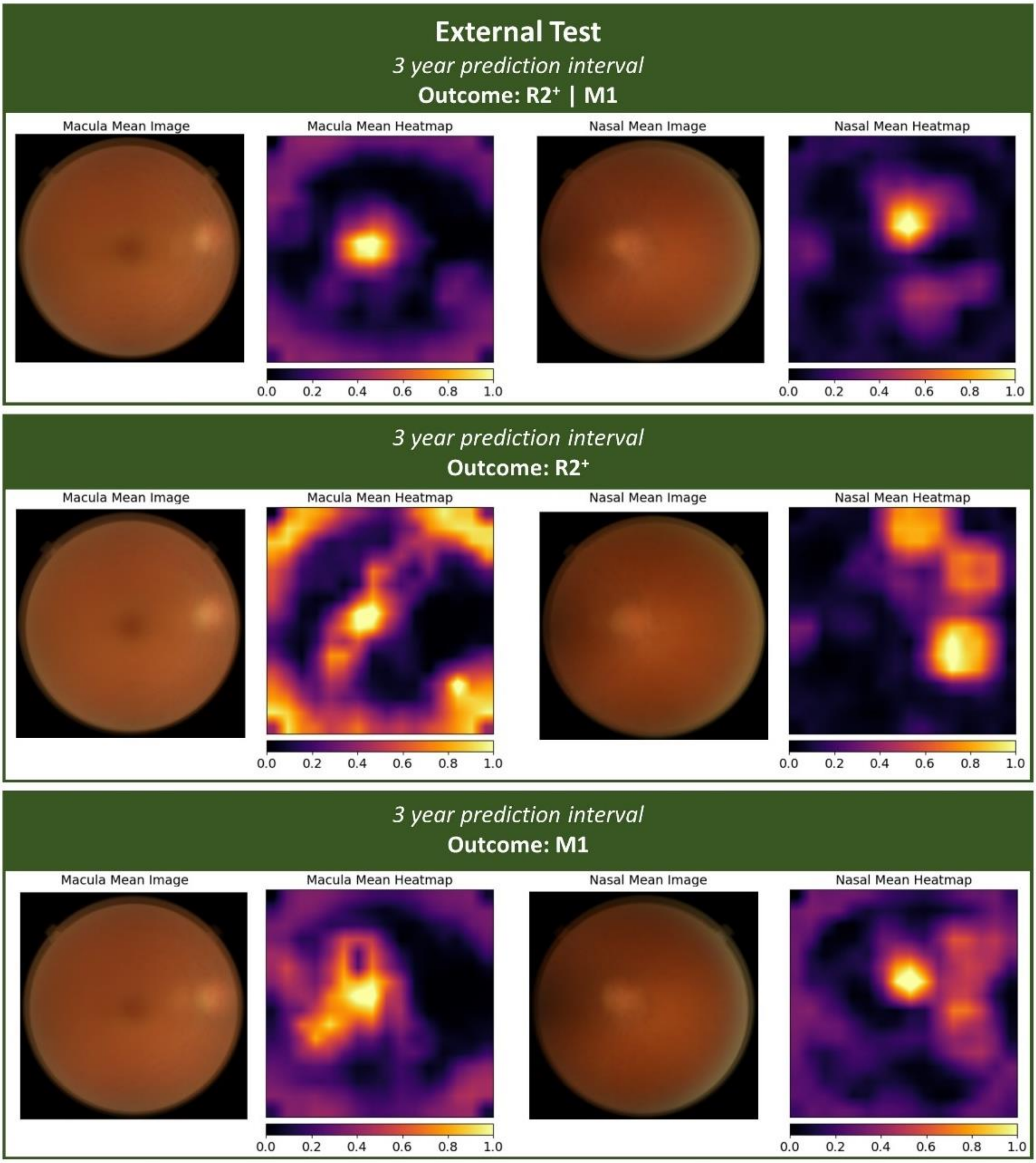


Mean heatmap computed using GradCAM[16] attribution method from 19 (BSBC-DESP) randomly sampled positive cases (*based on the ground truth*) for the 3-year prediction interval. Left macula and nasal fields are horizontally flipped to right eye orientation during pre-processing. Computation of mean images assumes approximate alignment on the basis of this pre-processing which is supported by fact that the fovea and optic disc remain distinguishable in the mean image. $R2^+$ | M1=Referable DR or maculopathy. $R2^+$=Referable DR. M1=Referable maculopathy.

## SUPPLEMENTARY FIGURE S16. SINGLE EYE IMAGE DLS ATTRIBUTIONS FOR THE 1 YEAR PREDICTION INTERVAL

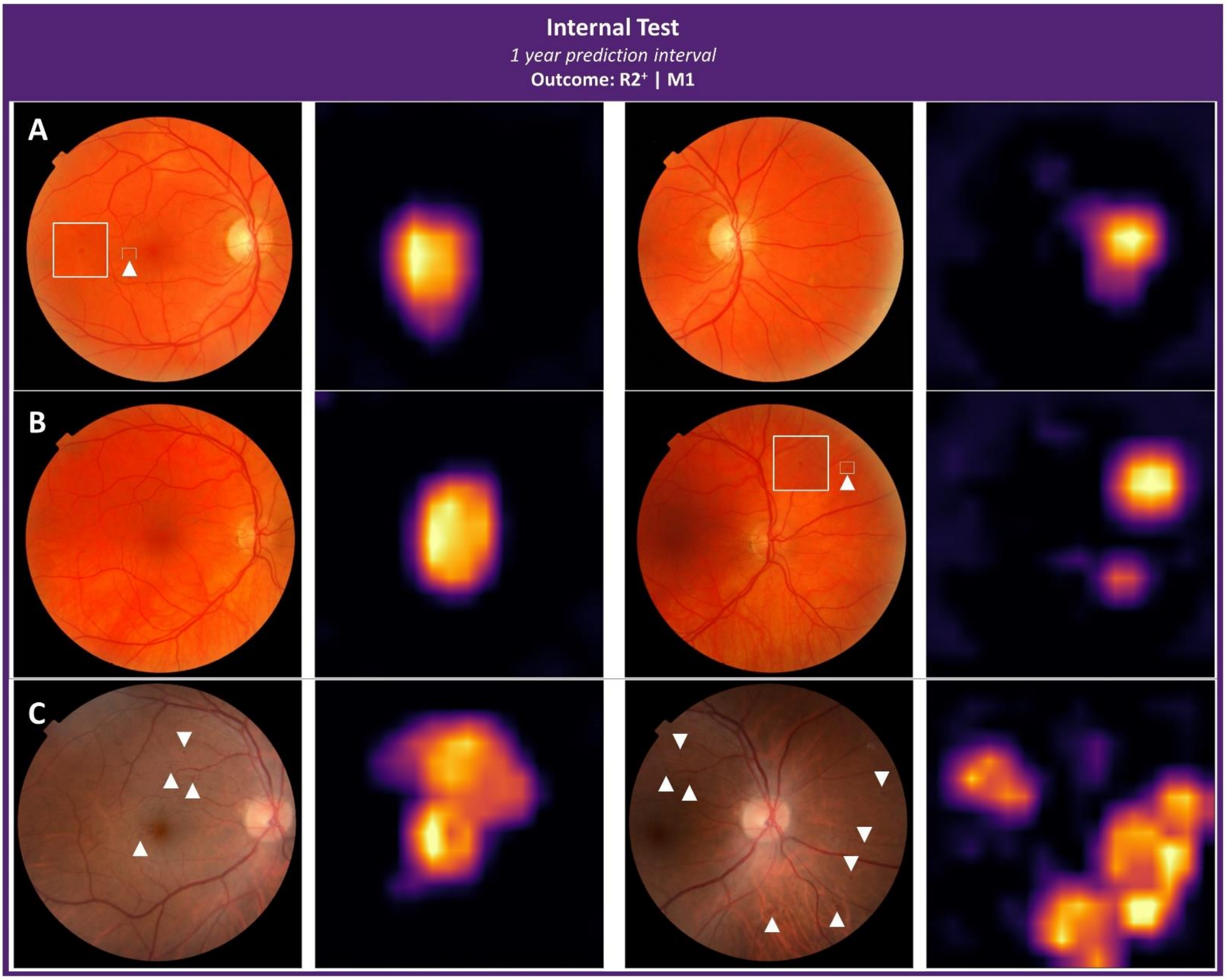


**Example A** shows highlighted central macula with a small microaneurysm temporal to the fovea (inset/arrow), and highlights an area temporal to the optic disc which has no clinically discernible DR lesions. **Example B** shows highlighted central macula with no clinically discernible DR lesions and an area temporal to the optic disc is also highlighted which has a small microaneurysm (inset/arrow). **Example C** shows multiple areas highlighted around the macula, as well as area superior-nasal and inferior-temporal to the optic disc which have multiple microaneurysms (arrows). GradCAM[16] attribution method for three example positive cases (*based on the ground truth*) for the 1-year prediction interval. Left macula and nasal fields are horizontally flipped to right eye orientation during pre-processing. $R2^+$ | M1=Referable DR or maculopathy.

## SUPPLEMENTARY FIGURE S17. SINGLE EYE IMAGE DLS ATTRIBUTIONS ANALYSIS FOR THE 2 YEAR PREDICTION INTERVAL

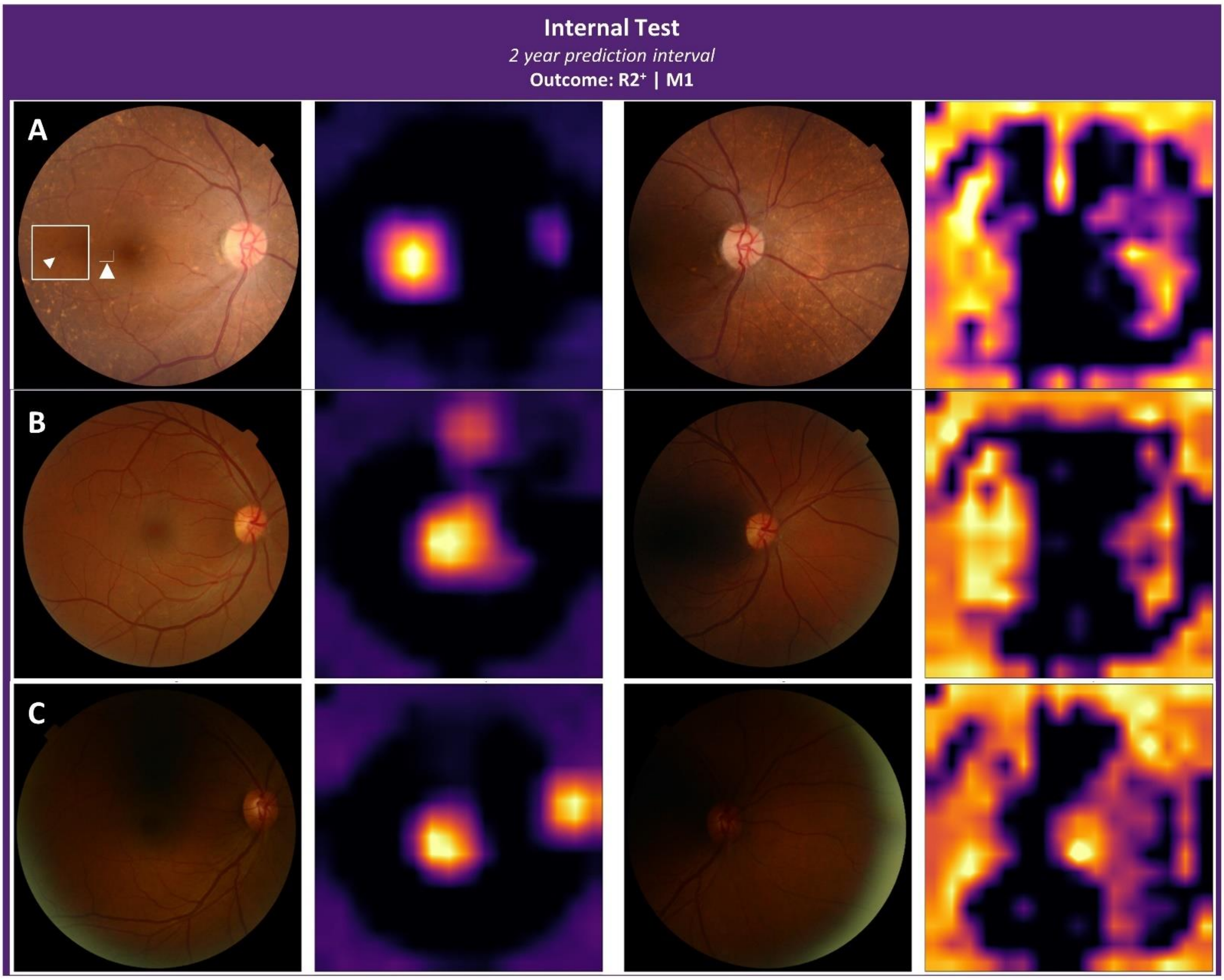


**Example A** shows highlighted central macula area with a small, barely discernible microaneurysm temporal to the fovea (inset/arrows), as well as multiple areas highlighted around the nasal field but with no clinically discernible DR lesions. **Example B** shows highlighted central macula area, and multiple areas highlighted around the nasal field with no clinically discernible DR lesions in either field although the nasal field image quality is limited. **Example C** shows highlighted central macula and optic disc, and multiple areas are also highlighted in the nasal field but no clinically discernible DR lesions are seen in either field, and additionally both fields have limited image quality. GradCAM[16] attribution method for three example positive cases (*based on the ground truth*) for the 2-year prediction interval. Left macula and nasal fields are horizontally flipped to right eye orientation during pre-processing. $R2^+$ | M1=Referable DR or maculopathy.

## SUPPLEMENTARY FIGURE S18. SINGLE EYE IMAGE DLS ATTRIBUTIONS ANALYSIS FOR THE 3 YEAR PREDICTION INTERVAL

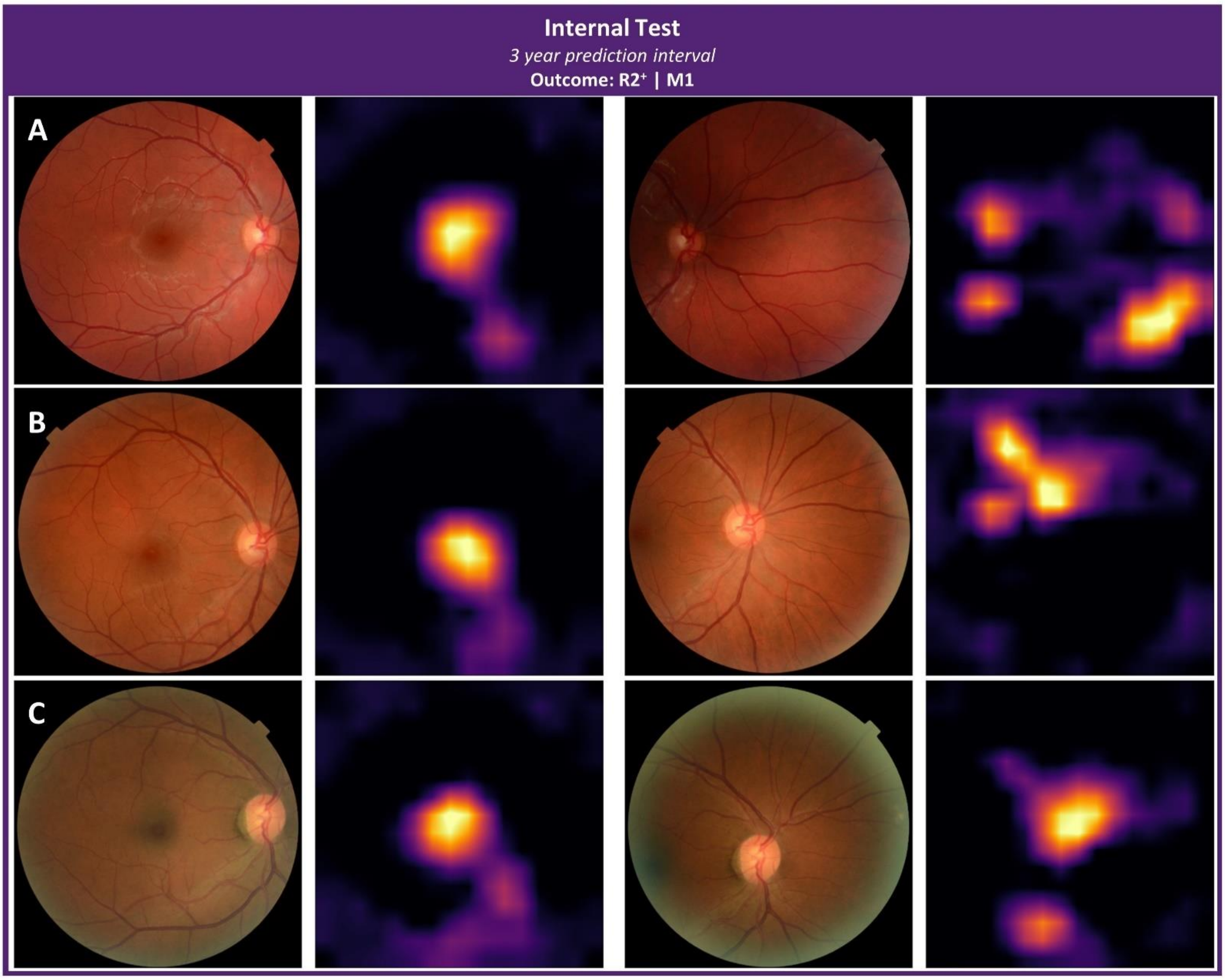


**Example A** shows highlighted central macula area, and areas highlighted temporal and far inferior-nasal to the optic disc with no clinically discernible DR lesions in either field. **Example B** shows highlighted central macula area, and an area highlighted superior-temporal to the optic disc but with no clinically discernible DR lesions in either field. **Example C** shows highlighted central macula area, and an area highlighted superior to the optic disc but with no clinically discernible DR lesions in either field. GradCAM[16] attribution method for three example positive cases (*based on the ground truth*) for the 3-year prediction interval. Left macula and nasal fields are horizontally flipped to right eye orientation during pre-processing. $R2^+$ | M1=Referable DR or maculopathy.

## SUPPLEMENTARY FIGURE S19. INTERNAL TEST TABULAR DLS ATTRIBUTIONS ANALYSIS FOR THE 1 YEAR PREDICTION INTERVAL

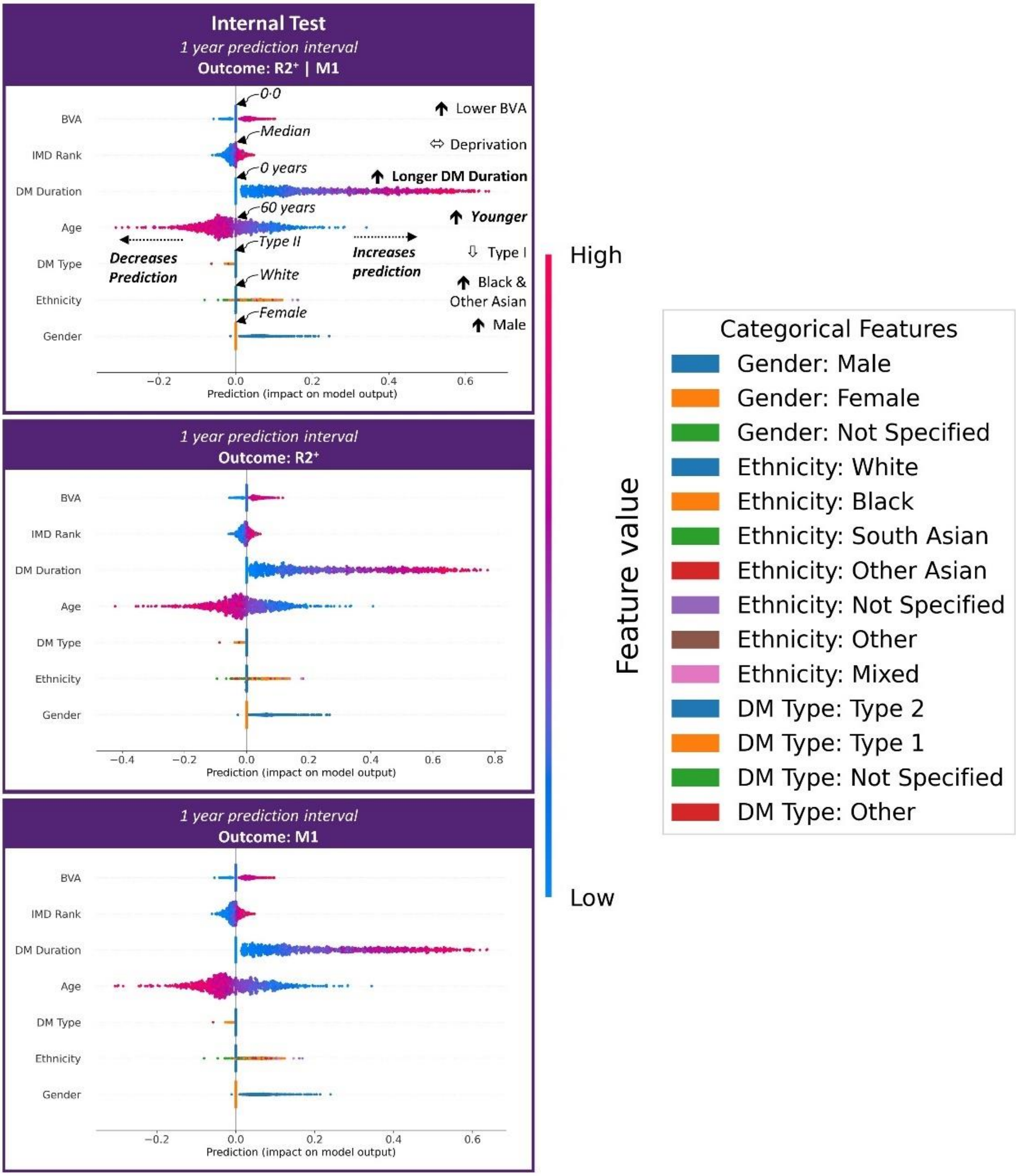


Integrated gradients[17] were used to compute tabular DLS attributions using 1,000 randomly drawn positive and negative cases for the 1-year prediction interval. The x-axis reflects the overall effect on model predictions of interpolating from the reference to case characteristic. Interpolation was performed in the embeddings space for categorical variables. Risk factor characteristics are coloured according to their value from blue to red for low to high values for continuous variables or a fixed value and colour for categorical variables. The reference for each risk factor characteristic, and an interpretation of how this characteristic interacts with tabular DLS predictions is shown in the first subplot. R2⁺ | M1=Referable DR or maculopathy. R2⁺=Referable DR. M1=Referable maculopathy. DM=Diabetes mellitus. BVA=Best visual acuity. IMD=Index of multiple deprivation.

## SUPPLEMENTARY FIGURE S20. EXTERNAL TEST TABULAR DLS ATTRIBUTIONS ANALYSIS FOR THE 1 YEAR PREDICTION INTERVAL

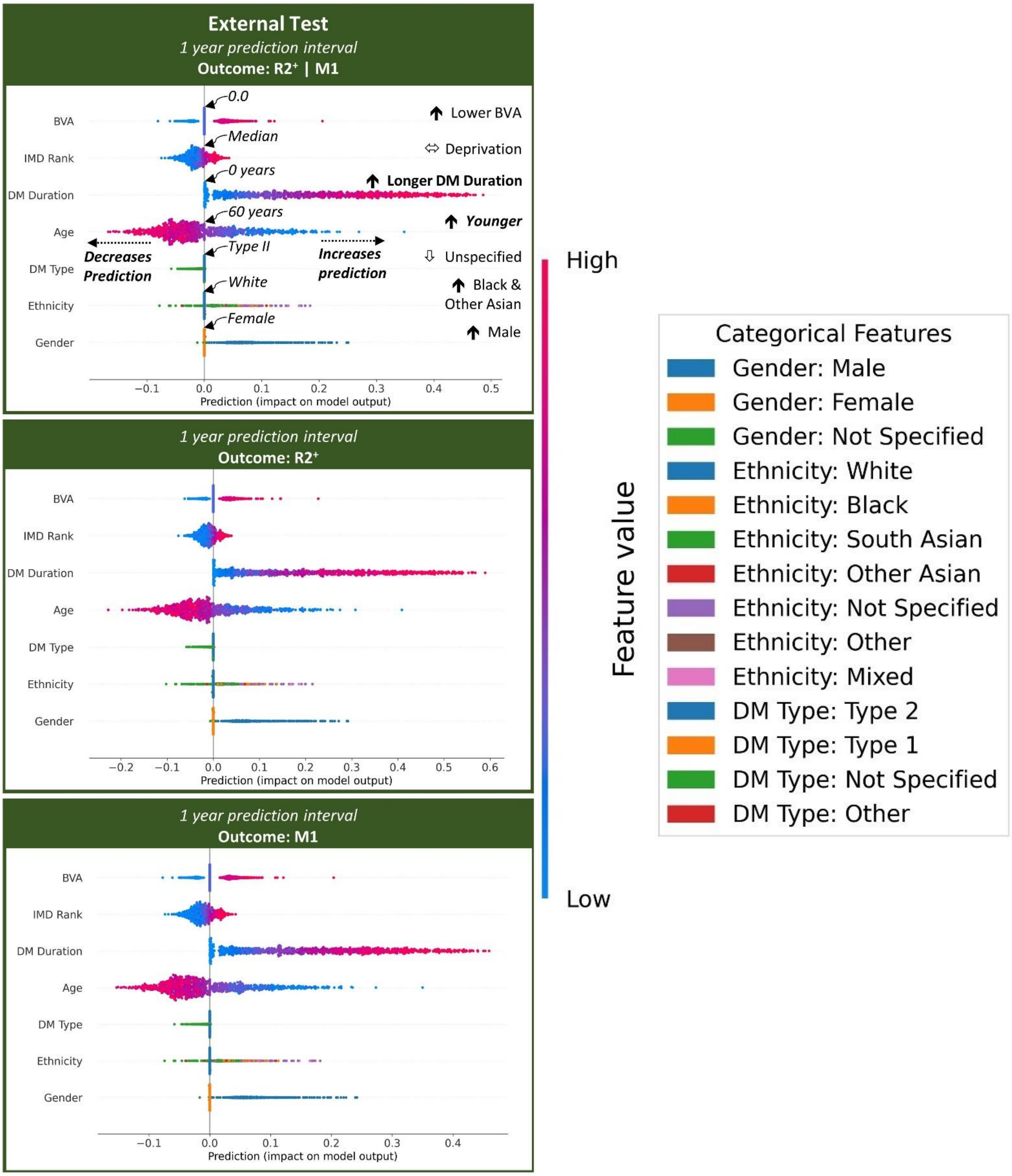


Integrated gradients[17] were used to compute DLS attributions using 1,000 randomly drawn positive and negative cases for the 1-year prediction interval. The x-axis reflects the overall effect on model predictions of interpolating from the reference to case characteristic. Interpolation was performed in the embeddings space for categorical variables. Risk factor characteristics are coloured according to their value from blue to red for low to high values for continuous variables or a fixed value and colour for categorical variables. The reference for each risk factor characteristic, and an interpretation of how this characteristic interacts with tabular DLS predictions is shown in the first subplot. $R2^+$ | M1=Referable DR or maculopathy. $R2^+$=Referable DR. M1=Referable maculopathy. DM=Diabetes mellitus. BVA=Best visual acuity. IMD=Index of multiple deprivation.

## SUPPLEMENTARY FIGURE S21. TABULAR DLS ATTRIBUTIONS ANALYSIS FOR THE 2 YEAR PREDICTION INTERVAL

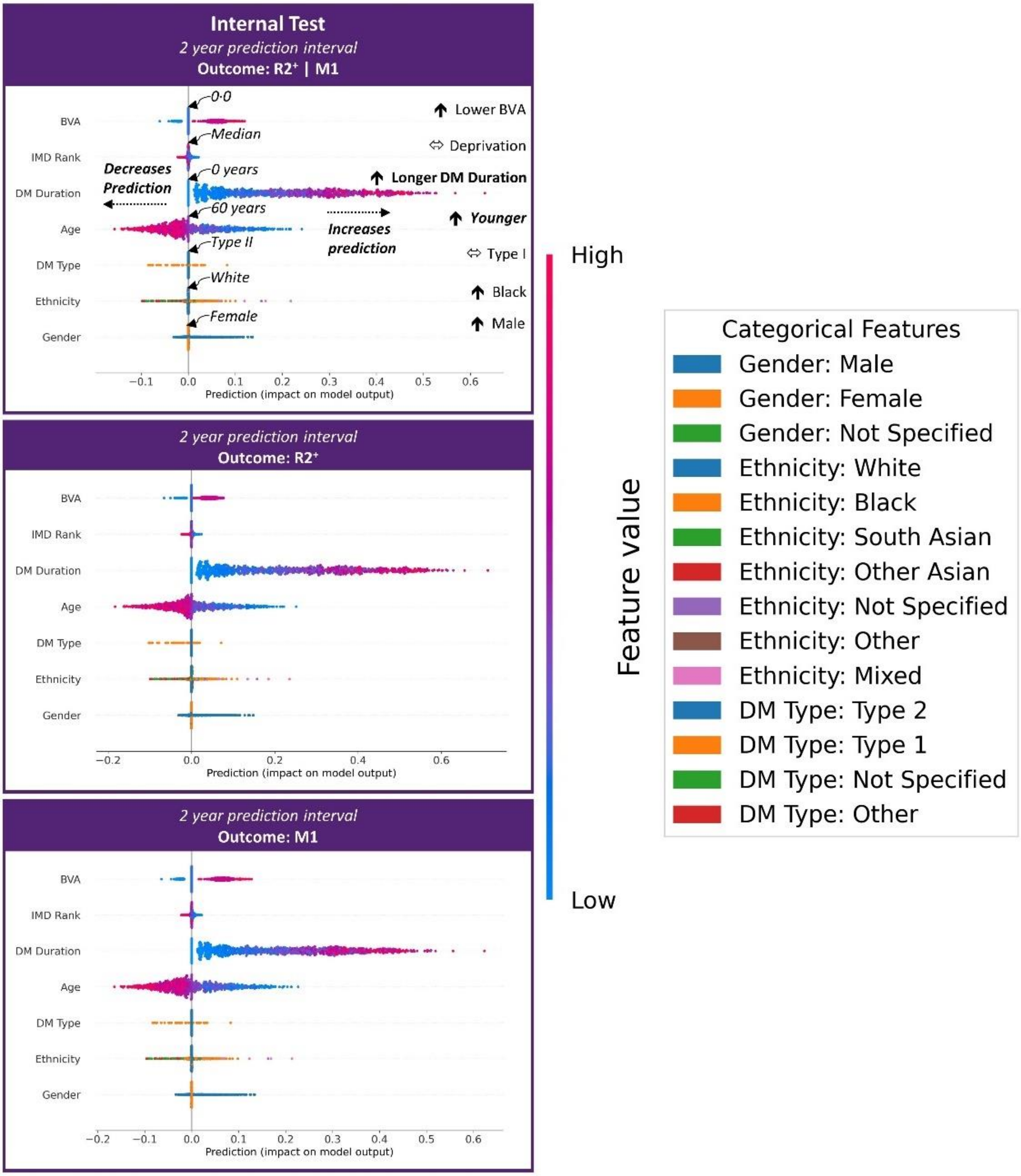


Integrated gradients[17] were used to compute tabular DLS attributions using 1,000 randomly drawn positive and negative cases for the 2-year prediction interval. The x-axis reflects the overall effect on model predictions of interpolating from the reference to case characteristic. Interpolation was performed in the embeddings space for categorical variables. Risk factor characteristics are coloured according to their value from blue to red for low to high values for continuous variables or a fixed value and colour for categorical variables. The reference for each risk factor characteristic, and an interpretation of how this characteristic interacts with tabular DLS predictions is shown in the first subplot. $R2^+$ | M1=Referable DR or maculopathy. $R2^+$=Referable DR. M1=Referable maculopathy. DM=Diabetes mellitus. BVA=Best visual acuity. IMD=Index of multiple deprivation.

## SUPPLEMENTARY FIGURE S22. EXTERNAL TEST TABULAR DLS ATTRIBUTIONS ANALYSIS FOR THE 1 YEAR PREDICTION INTERVAL

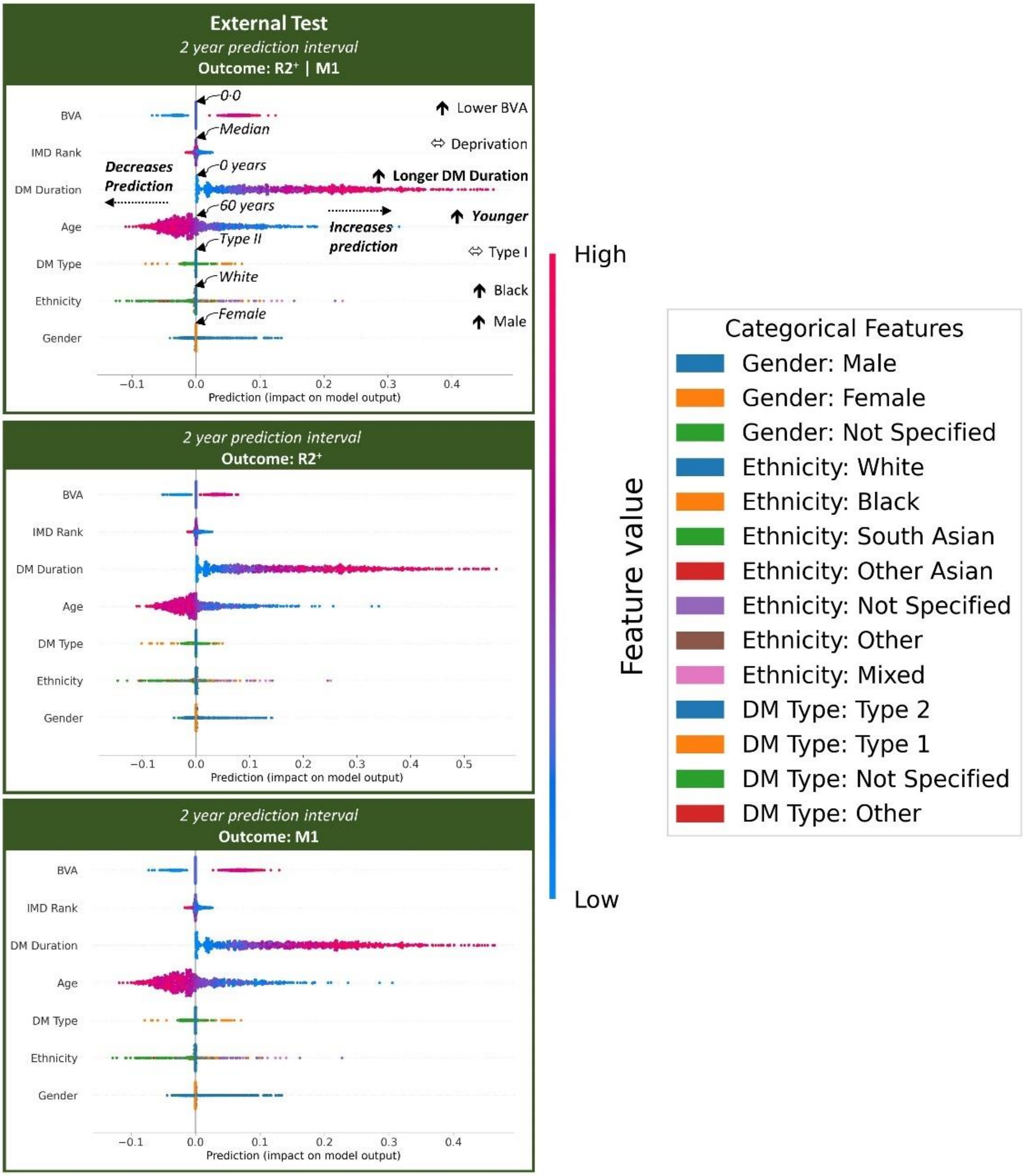


Integrated gradients[17] were used to compute tabular DLS attributions using 1,000 randomly drawn positive and negative cases for the 2-year prediction interval. The x-axis reflects the overall effect on model predictions of interpolating from the reference to case characteristic. Interpolation was performed in the embeddings space for categorical variables. Risk factor characteristics are coloured according to their value from blue to red for low to high values for continuous variables or a fixed value and colour for categorical variables. The reference for each risk factor characteristic, and an interpretation of how this characteristic interacts with tabular DLS predictions is shown in the first subplot. R2⁺ | M1=Referable DR or maculopathy. R2⁺=Referable DR. M1=Referable maculopathy. DM=Diabetes mellitus. BVA=Best visual acuity. IMD=Index of multiple deprivation.

## SUPPLEMENTARY FIGURE S23. TABULAR DLS ATTRIBUTIONS FOR THE 3 YEAR PREDICTION INTERVAL

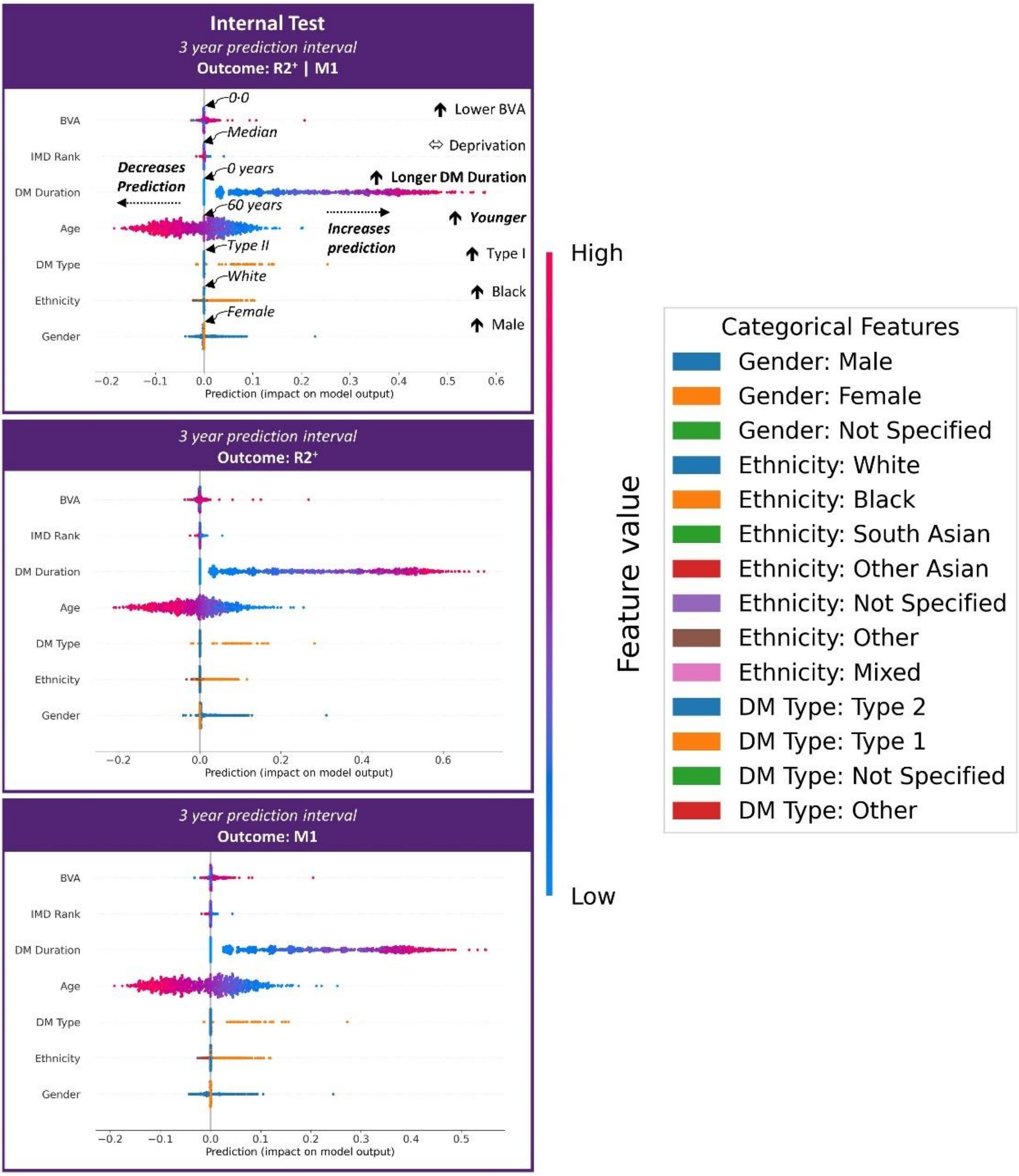


Integrated gradients[17] were used to compute tabular DLS attributions using 1,000 randomly drawn positive and negative cases for the 3-year prediction interval. The x-axis reflects the overall effect on model predictions of interpolating from the reference to case characteristic. Interpolation was performed in the embeddings space for categorical variables. Risk factor characteristics are coloured according to their value from blue to red for low to high values for continuous variables or a fixed value and colour for categorical variables. The reference for each risk factor characteristic, and an interpretation of how this characteristic interacts with tabular DLS predictions is shown in the first subplot. $R2^+$ | M1=Referable DR or maculopathy. $R2^+$=Referable DR. M1=Referable maculopathy. DM=Diabetes mellitus. BVA=Best visual acuity. IMD=Index of multiple deprivation.

## SUPPLEMENTARY FIGURE S24. EXTERNAL TEST TABULAR DLS ATTRIBUTIONS ANALYSIS FOR THE 1 YEAR PREDICTION INTERVAL

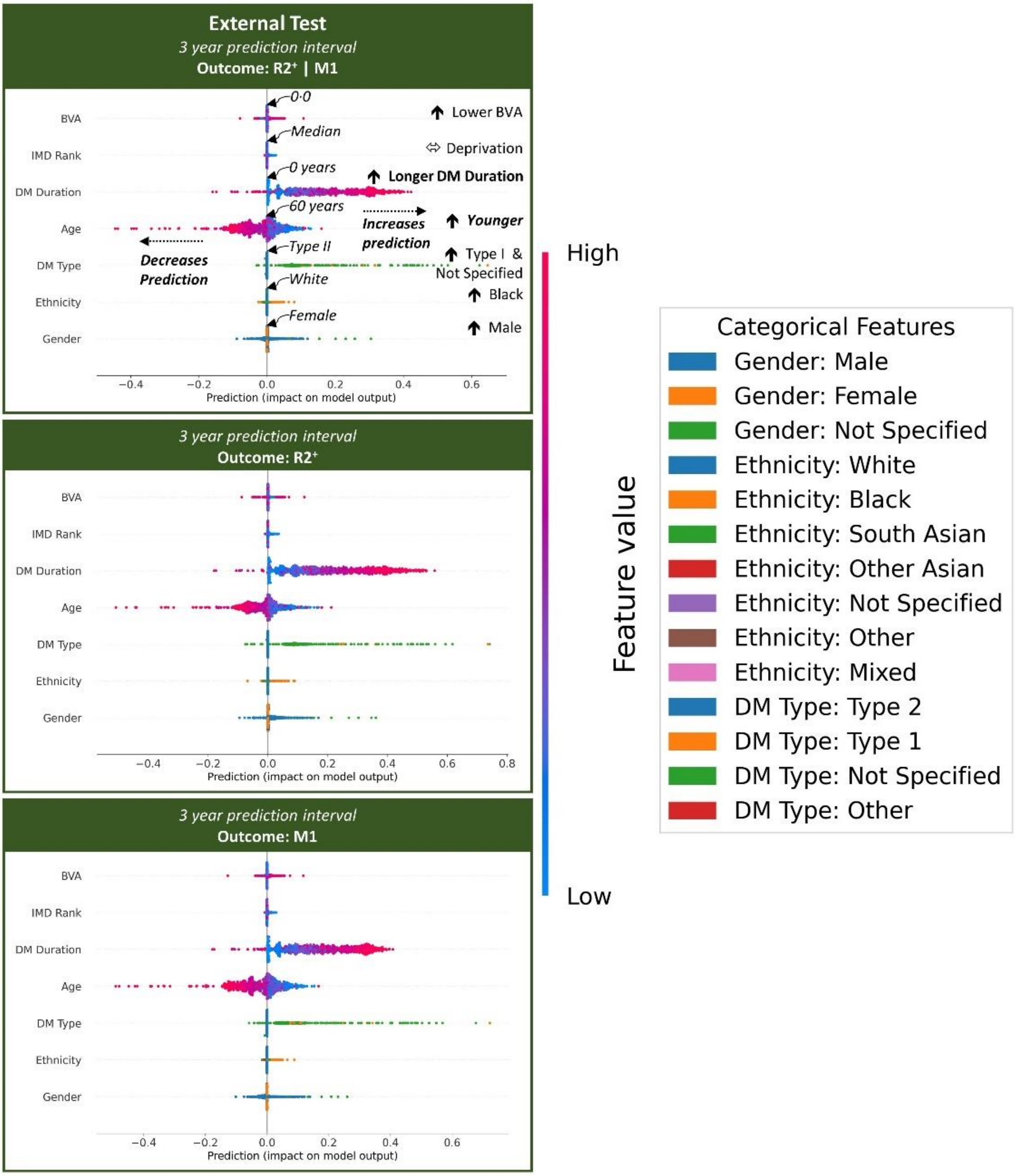


Integrated gradients[17] were used to compute tabular DLS attributions using 1,000 randomly drawn positive and negative cases for the 3-year prediction interval. The x-axis reflects the overall effect on model predictions of interpolating from the reference to case characteristic. Interpolation was performed in the embeddings space for categorical variables. Risk factor characteristics are coloured according to their value from blue to red for low to high values for continuous variables or a fixed value and colour for categorical variables. The reference for each risk factor characteristic, and an interpretation of how this characteristic interacts with tabular DLS predictions is shown in the first subplot. $R2^+$ | M1=Referable DR or maculopathy. $R2^+$=Referable DR. M1=Referable maculopathy. DM=Diabetes mellitus. BVA=Best visual acuity. IMD=Index of multiple deprivation.

# REFERENCES


1 PHE. *NHS Diabetic Eye Screening Programme: Grading definitions for referable disease*, <https://www.gov.uk/government/publications/diabetic-eye-screening-retinal-image-grading-criteria/nhs-diabetic-eye-screening-programme-grading-definitions-for-referable-disease> (2021).

2 PHE. *NHS public health functions agreement 2019-20: Service specification no.22 (NHS Diabetic Eye Screening Programme)*, <https://www.england.nhs.uk/wp-content/uploads/2017/04/Service-Specification-No.22-NHS_Diabetic_eye_screening.pdf> (2019).

3 PHE. *The management of grading quality: Good practice in the quality assurance of grading*, <https://assets.publishing.service.gov.uk/media/5a80521b40f0b62305b8a76c/The_Management_of_Grading.pdf> (2016).

4 McLennan, D. *et al.* The English Indices of Deprivation 2019. (Ministry of Housing, Communities and Local Government, 2019).

5 Murgatroyd, H. *et al.* Effect of mydriasis and different field strategies on digital image screening of diabetic eye disease. *The British journal of ophthalmology* **88**, 920-924, doi:10.1136/bjo.2003.026385 (2004).

6 PHE. *NHS Diabetic Eye Screening Programme: Overview of patient pathway, grading pathway, surveillance pathways and referral pathways*, <https://www.gov.uk/government/publications/diabetic-eye-screening-pathways-patient-grading-referral-surveillance> (2017).

7 Nderitu, P. *et al.* Automated image curation in diabetic retinopathy screening using deep learning. *Sci Rep* **12**, 11196, doi:10.1038/s41598-022-15491-1 (2022).

8 Kale, A. U. *et al.* A Datasheet for the INSIGHT Birmingham, Solihull and Black Country Diabetic Retinopathy Screening Dataset. *Ophthalmology Science*, doi:10.1016/j.xops.2023.100293.

9 Tan, M. & Le, Q. V. EfficientNetV2: Smaller Models and Faster Training. arXiv:2104.00298 (2021). <https://ui.adsabs.harvard.edu/abs/2021arXiv210400298T>.

10 Pak, A., Ziyaden, A., Tukeshev, K., Jaxylykova, A. & Abdullina, D. Comparative analysis of deep learning methods of detection of diabetic retinopathy. *Cogent Engineering* **7**, 1805144, doi:10.1080/23311916.2020.1805144 (2020).

11 Das, D., Biswas, S. K. & Bandyopadhyay, S. Detection of Diabetic Retinopathy using Convolutional Neural Networks for Feature Extraction and Classification (DRFEC). *Multimed Tools Appl*, 1-59, doi:10.1007/s11042-022-14165-4 (2022).

12 Arik, S. O. & Pfister, T. TabNet: Attentive Interpretable Tabular Learning. arXiv:1908.07442 (2019). <https://ui.adsabs.harvard.edu/abs/2019arXiv190807442A>.

13 Krause, J. *et al.* Grader Variability and the Importance of Reference Standards for Evaluating Machine Learning Models for Diabetic Retinopathy. *Ophthalmology* **125**, 1264-1272, doi:10.1016/j.ophtha.2018.01.034 (2018).

14 Eleuteri, A. *et al.* Individualised variable-interval risk-based screening for sight-threatening diabetic retinopathy: the Liverpool Risk Calculation Engine. *Diabetologia* **60**, 2174-2182, doi:10.1007/s00125-017-4386-0 (2017).

15 Haider, S. *et al.* Predictors for diabetic retinopathy progression-findings from nominal group technique and Evidence review. *BMJ Open Ophthalmol* **5**, e000579, doi:10.1136/bmjophth-2020-000579 (2020).

16 Selvaraju, R. R. *et al.* Grad-CAM: Visual Explanations from Deep Networks via Gradient-based Localization. arXiv:1610.02391 (2016). <https://ui.adsabs.harvard.edu/abs/2016arXiv161002391S>.

17 Sundararajan, M., Taly, A. & Yan, Q. Axiomatic Attribution for Deep Networks. arXiv:1703.01365 (2017). <https://ui.adsabs.harvard.edu/abs/2017arXiv170301365S>.

18 DeLong, E. R., DeLong, D. M. & Clarke-Pearson, D. L. Comparing the areas under two or more correlated receiver operating characteristic curves: a nonparametric approach. *Biometrics* **44**, 837-845 (1988).

19 Clopper, C. J. & Pearson, E. S. The use of confidence or fiducial limits illustrated in the case of the binomial. *Biometrika* **26**, 404-413, doi:10.1093/biomet/26.4.404 (1934).

# TRIPOD CHECKLIST: PREDICTION MODEL DEVELOPMENT AND VALIDATION

| Section/Topic | Item | | Checklist Item | Page |
|---|---|---|---|---|
| **Title and abstract** | | | | |
| Title | 1 | D;V | Identify the study as developing and/or validating a multivariable prediction model, the target population, and the outcome to be predicted. | 1 |
| Abstract | 2 | D;V | Provide a summary of objectives, study design, setting, participants, sample size, predictors, outcome, statistical analysis, results, and conclusions. | 2-3 |
| **Introduction** | | | | |
| Background and objectives | 3a | D;V | Explain the medical context (including whether diagnostic or prognostic) and rationale for developing or validating the multivariable prediction model, including references to existing models. | 4-5 |
| | 3b | D;V | Specify the objectives, including whether the study describes the development or validation of the model or both. | 4-5 |
| **Methods** | | | | |
| Source of data | 4a | D;V | Describe the study design or source of data (e.g., randomized trial, cohort, or registry data), separately for the development and validation data sets, if applicable. | 6 |
| | 4b | D;V | Specify the key study dates, including start of accrual; end of accrual; and, if applicable, end of follow-up. | 6 |
| Participants | 5a | D;V | Specify key elements of the study setting (e.g., primary care, secondary care, general population) including number and location of centres. | 6 |
| | 5b | D;V | Describe eligibility criteria for participants. | 6-7 |
| | 5c | D;V | Give details of treatments received, if relevant. | - |
| Outcome | 6a | D;V | Clearly define the outcome that is predicted by the prediction model, including how and when assessed. | 6-7 |
| | 6b | D;V | Report any actions to blind assessment of the outcome to be predicted. | - |
| Predictors | 7a | D;V | Clearly define all predictors used in developing or validating the multivariable prediction model, including how and when they were measured. | 6-8 |
| | 7b | D;V | Report any actions to blind assessment of predictors for the outcome and other predictors. | - |
| Sample size | 8 | D;V | Explain how the study size was arrived at. | 6 |
| Missing data | 9 | D;V | Describe how missing data were handled (e.g., complete-case analysis, single imputation, multiple imputation) with details of any imputation method. | 8 |
| Statistical analysis methods | 10a | D | Describe how predictors were handled in the analyses. | 7-9 |
| | 10b | D | Specify type of model, all model-building procedures (including any predictor selection), and method for internal validation. | 7-9 |
| | 10c | V | For validation, describe how the predictions were calculated. | 7-9 |
| | 10d | D;V | Specify all measures used to assess model performance and, if relevant, to compare multiple models. | 8-9 |
| | 10e | V | Describe any model updating (e.g., recalibration) arising from the validation, if done. | - |
| Risk groups | 11 | D;V | Provide details on how risk groups were created, if done. | 7 |
| Development vs. validation | 12 | V | For validation, identify any differences from the development data in setting, eligibility criteria, outcome, and predictors. | 6-7 |
| **Results** | | | | |
| Participants | 13a | D;V | Describe the flow of participants through the study, including the number of participants with and without the outcome and, if applicable, a summary of the follow-up time. A diagram may be helpful. | 9 |
| | 13b | D;V | Describe the characteristics of the participants (basic demographics, clinical features, available predictors), including the number of participants with missing data for predictors and outcome. | 9 |
| | 13c | V | For validation, show a comparison with the development data of the distribution of important variables (demographics, predictors, and outcome). | 9-10 |
| Model development | 14a | D | Specify the number of participants and outcome events in each analysis. | 9-10 |
| | 14b | D | If done, report the unadjusted association between each candidate predictor and outcome. | 9-10 |
| Model specification | 15a | D | Present the full prediction model to allow predictions for individuals (i.e., all regression coefficients, and model intercept or baseline survival at a given time point). | 9-11 |
| | 15b | D | Explain how to the use the prediction model. | 9-11 |
| Model performance | 16 | D;V | Report performance measures (with CIs) for the prediction model. | 9-11 |
| Model-updating | 17 | V | If done, report the results from any model updating (i.e., model specification, model performance). | - |
| **Discussion** | | | | |
| Limitations | 18 | D;V | Discuss any limitations of the study (such as nonrepresentative sample, few events per predictor, missing data). | 15 |
| Interpretation | 19a | V | For validation, discuss the results with reference to performance in the development data, and any other validation data. | 12-14 |
| | 19b | D;V | Give an overall interpretation of the results, considering objectives, limitations, results from similar studies, and other relevant evidence. | 12-14 |
| Implications | 20 | D;V | Discuss the potential clinical use of the model and implications for future research. | 12-14 |
| **Other information** | | | | |
| Supplementary information | 21 | D;V | Provide information about the availability of supplementary resources, such as study protocol, Web calculator, and data sets. | 16 |
| Funding | 22 | D;V | Give the source of funding and the role of the funders for the present study. | 17 |
