# Supplementary material for: Predicting 1, 2 and 3 year emergent referable diabetic retinopathy and maculopathy using deep learning
Source: Commun Med (Lond). 2024 Aug 21;4:167. doi: 10.1038/s43856-024-00590-z (PMC11339445; doi:10.1038/s43856-024-00590-z)
Supplement: Supplementary file 3 — Description of Additional Supplementary Files [file 43856_2024_590_MOESM3_ESM.pdf]

Description of Additional Supplementary Files

File name: Supplementary Data 1

File description: Cohorts for the 1, 2 and 3 year prediction intervals are created from the 'Longitudinal Datasets' and described

File name: Supplementary Data 2 - 4

File description: Subgroup analysis whereby DLS performance was stratified by age, sex and ethnicity were performed, with results
