# Supplementary material for: Predicting 1, 2 and 3 year emergent referable diabetic retinopathy and maculopathy using deep learning
Source: Commun Med (Lond). 2024 Aug 21;4:167. doi: 10.1038/s43856-024-00590-z (PMC11339445; doi:10.1038/s43856-024-00590-z)
Supplement: Supplementary file 4 — Supplementary Data 1 [file 43856_2024_590_MOESM4_ESM.docx]

# Supplementary Data 1. Longitudinal dataset characteristics

| Characteristic | | SEL-DESP | | | | | | | | | BSBC-DESP | | |
| --- | --- | --- | --- | --- | --- | --- | --- | --- | --- | --- | --- | --- | --- |
|  |  | Development: Longitudinal Dataset  110,837 unique eyes, 55,610 individuals^1^ | | | | | | Internal Test: Longitudinal Dataset  27,996 unique eyes  14,047 individuals^1^ | | | External Test: Longitudinal Dataset  6,928 unique eyes  3,490 individuals^1^ | | |
|  |  | Train (88%) | | | Tune (12%) | | |  |  |  |  |  |  |
|  |  | N (%) or [mean] (SD) | | | N (%) or [mean] (SD) | | | N (%) or [mean] (SD) | | | N (%) or [mean] (SD) | | |
|  |  | Year 1 | Year 2 | Year 3 | Year 1 | Year 2 | Year 3 | Year 1 | Year 2 | Year 3 | Year 1 | Year 2 | Year 3 |
| Unique Eyes  Individuals | | 87,328  44,437 | 62,683  32,015 | 49,985  25,627 | 11,936  6,060 | 8,577  4,366 | 6,813  3,495 | 25,154  12,800 | 18,100  9,228 | 14,327  7,345 | 6,355  3,264 | 5,506  2,834 | 4,865  2,519 |
| Age | Years | [62] (14) | [61] (14) | [60] (14) | [63] (14) | [61] (14) | [61] (13) | [62] (14) | [62] (14) | [64] (14) | [64] (14) | [64] (14) | [63] (13) |
| Sex | Male | 47,520  (54.4) | 33,963  (54.2) | 26,575  (53.2) | 6,445  (54.0) | 4,581  (53.4) | 3,393  (52.7) | 13,610  (54.1) | 9,827  (54.3) | 7,626  (53.2) | 3,264  (51.4) | 2,810  (51.0) | 2,489  (51.2) |
|  | Female | 39,808  (45.6) | 28,720  (45.8) | 23,410  (46.8) | 5,491  (46.0) | 3,996  (46.6) | 3,220  (47.3) | 11,541  (45.9) | 8,271  (45.7) | 6,701  (46.8) | 2,982  (46.9) | 2,619  (47.6) | 2,301  (47.3) |
|  | Not Specified | 0  (0.0) | 0  (0.0) | 0  (0.0) | 0  (0.0) | 0  (0.0) | 0  (0.0) | 3  (<0.1) | 2  (<0.1) | 0  (0.0) | 109  (1.7) | 77  (1.4) | 75  (1.5) |
| Ethnicity | White | 46,931  (53.7) | 33,260  (53.1) | 24,554  (49.1) | 6,273  (53.6) | 4,455  (51.9) | 3,391  (49.8) | 13,622  (54.2) | 9,753  (53.9) | 7,168  (50.0) | 3,706  (58.3) | 3,290  (59.8) | 2,900  (59.6) |
|  | Black | 23,085  (26.4) | 17,132  (27.3) | 15,640  (31.3) | 3,351  (28.1) | 2,493  (29.1) | 2,145  (31.5) | 6,715  (26.7) | 4,908  (27.1) | 4,464  (31.2) | 376  (5.9) | 312  (5.7) | 290  (6.0) |
|  | South Asian | 5,595  (6.5) | 4,071  (6.5) | 3,169  (6.3) | 742  (6.2) | 584  (6.8) | 390  (5.7) | 1,647  (6.6) | 1,210  (6.7) | 924  (6.5) | 1,453  (22.9) | 1,256  (22.8) | 1,125  (23.1) |
|  | Other Asian | 5,659  (6.5) | 4,228  (6.8) | 3,301  (6.6) | 777  (6.5) | 478  (5.6) | 438  (6.4) | 1,608  (6.4) | 1,182  (6.5) | 889  (6.2) | 125  (2.0) | 110  (2.0) | 104  (2.1) |
|  | Mixed | 2,317  (2.7) | 1,601  (2.6) | 1,348  (2.7) | 308  (2.6) | 237  (2.8) | 181  (2.7) | 561  (2.3) | 400  (2.2) | 339  (2.4) | 78  (1.2) | 56  (1.0) | 43  (0.9) |
|  | Other | 2,294  (2.6) | 1,555  (2.5) | 1,310  (2.6) | 307  (2.6) | 224  (2.6) | 185  (2.7) | 586  (2.3) | 418  (2.3) | 328  (2.3) | 50  (0.8) | 34  (0.6) | 27  (0.6) |
|  | Not Specified | 1,447  (1.7) | 836  (1.3) | 663  (1.3) | 178  (1.5) | 106  (1.2) | 83  (1.2) | 415  (1.7) | 229  (1.3) | 215  (1.5) | 567  (8.9) | 448  (8.1) | 376  (7.7) |
| Visual Acuity | LogMAR | [0.14]  (0.23) | [0.13]  (0.22) | [0.15]  (0.21) | [0.14]  (0.24) | [0.14]  (0.23) | [0.15]  (0.21) | [0.14]  (0.23) | [0.13]  (0.21) | [0.15]  (0.21) | [0.11]  (0.23) | [0.10]  (0.22) | [0.08]  (0.19) |
| IMD | Rank | [13,712]  (7,991) | [13,522]  (7,908) | [12,775]  (7,462) | [13,668]  (7,941) | [13,418]  (7,876) | [12,702]  (7,514) | [13,675]  (7,963) | [13,459]  (7,860) | [12,772]  (7,398) | [9,834]  (8,837) | [9,904]  (8,831) | [9,982]  (8,914) |
| DM Type | Type 2 | 82,378  (94.3) | 59,308  (94.6) | 47,335  (94.7) | 11,340  (95.0) | 8,083  (94.2) | 6,484  (95.2) | 23,773  (94.5) | 17,104  (94.5) | 13,564  (94.7) | 3,734  (58.8) | 3,299  (59.9) | 2,969  (61.0) |
|  | Type 1 | 4,727  (5.4) | 3,272  (5.2) | 2,574  (5.2) | 572  (4.8) | 480  (5.6) | 319  (4.7) | 1,313  (5.2) | 968  (5.4) | 740  (5.2) | 183  (2.9) | 160  (2.9) | 143  (2.9) |
|  | Other | 111  (0.1) | 66  (0.1) | 55  (0.1) | 10  (<0.1) | 8  (0.1) | 4  (<0.1) | 30  (0.1) | 13  (<0.1) | 10  (<0.1) | 8  (<0.1) | 3  (<0.1) | 3  (<0.1) |
|  | Not Specified | 112  (0.2) | 37  (0.1) | 21  (<0.1) | 14  (0.1) | 6  (0.1) | 6  (<0.1) | 38  (0.2) | 15  (<0.1) | 13  (<0.1) | 2,430  (38.2) | 2,044  (37.1) | 1,750  (36.0) |
| DM Duration | Years | [8.5] (7.1) | [7.9] (6.8) | [7.6] (6.8) | [8.5] (7.2) | [8.5] (7.2) | [7.5] (6.8) | [8.5] (7.1) | [8.0] (6.9) | [7.6] (6.8) | [6.4] (3.8) | [6.0] (3.5) | [5.5] (3.2) |
| Incident Referable DR | R2^+^ | 335  (0.38) | 212  (0.34) | 216  (0.43) | 31  (0.26) | 25  (0.29) | 28  (0.41) | 70  (0.28) | 43  (0.24) | 62  (0.43) | 19  (0.30) | 21  (0.38) | 21  (0.43) |
| Incident Referable Maculopathy | M1 | 1,462  (1.67) | 1,019  (1.63) | 1,024  (2.05) | 197  (1.65) | 148  (1.73) | 146  (2.14) | 437  (1.74) | 308  (1.70) | 296  (2.07) | 130  (2.05) | 95  (1.73) | 110  (2.26) |

^1^Numbers describe the overall number of unique eyes and individuals between the 1, 2 and 3 year longitudinal cohorts. Individuals/eyes can be in all prediction intervals but only exclusively in one partition (train, tune or text). SEL-DESP=South-east London diabetic eye screening programme. BSBC-DESP=Birmingham, Solihull and Black Country diabetic eye screening programme. SD=Standard deviation. LogMAR=Logarithm of the minimal angle of resolution. IMD=Index of multiple deprivation. DM=Diabetes mellitus. DR=Diabetic retinopathy. R2^+^=Referable DR. M1=Referable maculopathy.
