# Supplementary material for: Predicting 1, 2 and 3 year emergent referable diabetic retinopathy and maculopathy using deep learning
Source: Commun Med (Lond). 2024 Aug 21;4:167. doi: 10.1038/s43856-024-00590-z (PMC11339445; doi:10.1038/s43856-024-00590-z)
Supplement: Supplementary file 6 — Supplementary Data 3 [file 43856_2024_590_MOESM6_ESM.docx]

# Supplementary Data 3. Image DLS performance stratified by demographics

| Strata | | Outcome | Internal Test  AUROC (95%CI) | | | | | | | | | External Test  AUROC (95%CI) | | | | | | | | |
| --- | --- | --- | --- | --- | --- | --- | --- | --- | --- | --- | --- | --- | --- | --- | --- | --- | --- | --- | --- | --- |
|  |  |  | Test Size | +ve | Year 1 | Test Size | +ve | Year 2 | Test Size | +ve | Year 3 | Test Size | +ve | Year 1 | Test Size | +ve | Year 2 | Test Size | +ve | Year 3 |
| All | | R2^+^ \| M1 | 25,154 | 477 | 0.84  (0.82-0.86) | 18,100 | 331 | 0.82  (0.80-0.85) | 14,327 | 327 | 0.76  (0.74-0.79) | 6,335 | 143 | 0.84  (0.82-0.89) | 5,506 | 107 | 0.78  (0.73-0.84) | 4,865 | 118 | 0.77  (0.72-0.82) |
|  |  | R2^+^ | 25,154 | 70 | 0.96  (0.93-0.98) | 18,100 | 43 | 0.90  (0.85-0.95) | 14,327 | 62 | 0.82  (0.77-0.88) | 6,335 | 19 | 0.93  (0.90-0.97) | 5,506 | 21 | 0.90  (0.83-0.97) | 4,865 | 21 | 0.85  (0.74-0.97) |
|  |  | M1 | 25,154 | 437 | 0.83  (0.81-0.85) | 18,100 | 308 | 0.82  (0.79-0.85) | 14,327 | 296 | 0.76  (0.73-0.79) | 6,335 | 130 | 0.85  (0.81-0.89) | 5,506 | 95 | 0.78  (0.72-0.83) | 4,865 | 110 | 0.77  (0.72-0.82) |
| Age Group | ≤40y | R2^+^ \| M1 | 1,574 | 49 | 0.83  (0.75-0.91) | 1,188 | 46 | 0.84  (0.79-0.90) | 989 | 48 | 0.73  (0.66-0.81) | 376 | 15 | 0.89  (0.80-0.98) | 284 | 12 | 0.78  (0.66-0.90) | 272 | 13 | 0.52  (0.32-0.71) |
|  |  | R2^+^ | 1,574 | 8 | 0.95  (0.90-1.00) | 1,188 | 5 | 0.87  (0.74-1.00) | 989 | 9 | 0.73  (0.54-0.92) | 376 | 3 | 0.90  (0.73-1.00) | 284 | 5 | 0.76  (0.56-0.97) | 272 | 2 | 0.29  (0.23-0.34) |
|  |  | M1 | 1,574 | 44 | 0.82  (0.74-0.90) | 1,188 | 44 | 0.84  (0.79-0.90) | 989 | 44 | 0.74  (0.67-0.82) | 376 | 14 | 0.89  (0.77-0.90) | 284 | 10 | 0.82  (0.69-0.84) | 272 | 13 | 0.51  (0.31-0.70) |
|  | 41-70y | R2^+^ \| M1 | 16,032 | 344 | 0.85  (0.83-0.88) | 12,118 | 248 | 0.85  (0.83-0.88) | 9,779 | 247 | 0.75  (0.71-0.78) | 3,673 | 100 | 0.87  (0.83-0.91) | 3,362 | 73 | 0.81  (0.75-0.87) | 3,079 | 81 | 0.78  (0.72-0.84) |
|  |  | R2^+^ | 16,032 | 57 | 0.97  (0.94-0.99) | 12,118 | 37 | 0.97  (0.94-0.99) | 9,779 | 48 | 0.83  (0.77-0.90) | 3,673 | 14 | 0.92  (0.87-0.97) | 3,362 | 13 | 0.90  (0.80-1.00) | 3,079 | 17 | 0.94  (0.86-1.00) |
|  |  | M1 | 16,032 | 312 | 0.84  (0.81-0.87) | 12,118 | 228 | 0.84  (0.81-0.87) | 9,779 | 224 | 0.74  (0.81-0.78) | 3,673 | 90 | 0.87  (0.82-0.91) | 3,362 | 65 | 0.80  (0.74-0.87) | 3,079 | 75 | 0.77  (0.70-0.84) |
|  | ≥71y | R2^+^ \| M1 | 7,548 | 84 | 0.79  (0.74-0.85) | 4,794 | 37 | 0.78  (0.70-0.87) | 3,559 | 32 | 0.78  (0.71-0.86) | 2,306 | 28 | 0.76  (0.64-0.88) | 1,860 | 22 | 0.67  (0.52-0.81) | 1,514 | 24 | 0.78  (0.66-0.90) |
|  |  | R2^+^ | 7,548 | 5 | 0.86  (0.71-1.00) | 4,794 | 1 | 0.78  (*) | 3,559 | 5 | 0.82  (0.69-0.94) | 2,306 | 2 | 0.99  (0.98-1.00) | 1,860 | 3 | 0.99  (0.98-1.00) | 1,514 | 2 | 0.50  (0.00-1.00) |
|  |  | M1 | 7,548 | 81 | 0.79  (0.73-0.84) | 4,794 | 36 | 0.78  (0.69-0.87) | 3,559 | 28 | 0.78  (0.70-0.87) | 2,306 | 26 | 0.74  (0.62-0.86) | 1,860 | 20 | 0.64  (0.49-0.83) | 1,514 | 22 | 0.81  (0.71-0.91) |
| Sex | M | R2^+^ \| M1 | 13,610 | 271 | 0.87  (0.85-0.90) | 9,827 | 191 | 0.83  (0.80-0.86) | 7,626 | 183 | 0.77  (0.73-0.80) | 3,264 | 85 | 0.86  (0.82-0.91) | 2,810 | 63 | 0.78  (0.71-0.84) | 2,489 | 68 | 0.79  (0.72-0.86) |
|  |  | R2^+^ | 13,610 | 51 | 0.98  (0.97-0.99) | 9,827 | 27 | 0.93  (0.88-0.98) | 7,626 | 43 | 0.82  (0.75-0.89) | 3,264 | 11 | 0.90  (0.84-0.96) | 2,810 | 12 | 0.86  (0.75-0.97) | 2,489 | 11 | 0.82  (0.63-1.00) |
|  |  | M1 | 13,610 | 240 | 0.86  (0.83-0.89) | 9,827 | 177 | 0.83  (0.80-0.86) | 7,626 | 161 | 0.77  (0.73-0.81) | 3,264 | 74 | 0.85  (0.80-0.90) | 2,810 | 54 | 0.77  (0.70-0.84) | 2,489 | 62 | 0.80  (0.73-0.87) |
|  | F | R2^+^ \| M1 | 11,541 | 209 | 0.80  (0.76-0.84) | 8,271 | 140 | 0.81  (0.77-0.86) | 6,701 | 144 | 0.76  (0.72-0.81) | 2,982 | 58 | 0.84  (0.78-0.91) | 2,619 | 44 | 0.80  (0.71-0.88) | 2,301 | 49 | 0.76  (0.68-0.83) |
|  |  | R2^+^ | 11,541 | 19 | 0.89  (0.80-0.99) | 8,271 | 16 | 0.85  (0.74-0.96) | 6,701 | 19 | 0.82  (0.73-0.92) | 2,982 | 8 | 0.98  (0.95-1.00) | 2,619 | 9 | 0.97  (0.93-1.00) | 2,301 | 10 | 0.88  (0.76-1.00) |
|  |  | M1 | 11,541 | 197 | 0.79  (0.75-0.83) | 8,271 | 131 | 0.81  (0.76-0.85) | 6,701 | 135 | 0.76  (0.71-0.80) | 2,982 | 56 | 0.84  (0.77-0.90) | 2,619 | 41 | 0.79  (0.69-0.88) | 2,301 | 47 | 0.75  (0.67-0.82) |
| Ethnicity | White | R2^+^ \| M1 | 13,622 | 203 | 0.85  (0.82-0.89) | 9,753 | 130 | 0.85  (0.81-0.89) | 7,168 | 105 | 0.78  (0.73-0.83) | 3,706 | 62 | 0.87  (0.82-0.92) | 3,290 | 56 | 0.86  (0.80-0.92) | 2,900 | 54 | 0.83  (0.76-0.90) |
|  |  | R2^+^ | 13,622 | 34 | 0.96  (0.93-1.00) | 9,753 | 16 | 0.91  (0.85-0.98) | 7,168 | 20 | 0.82  (0.71-0.93) | 3,706 | 9 | 0.95  (0.91-0.98) | 3,290 | 10 | 0.96  (0.92-0.99) | 2,900 | 10 | 0.82  (0.60-1.00) |
|  |  | M1 | 13,622 | 186 | 0.84  (0.81-0.88) | 9,753 | 120 | 0.84  (0.80-0.88) | 7,168 | 96 | 0.78  (0.73-0.83) | 3,706 | 55 | 0.86  (0.80-0.92) | 3,290 | 50 | 0.86  (0.79-0.92) | 2,900 | 49 | 0.84  (0.77-0.98) |
|  | Black | R2^+^ \| M1 | 6,715 | 176 | 0.81  (0.78-0.85) | 4,908 | 129 | 0.80  (0.75-0.84) | 4,464 | 155 | 0.74  (0.69-0.78) | 376 | 12 | 0.76  (0.59-0.93) | 312 | 4 | 0.57  (0.19-0.95) | 290 | 9 | 0.88  (0.78-0.98) |
|  |  | R2^+^ | 6,715 | 20 | 0.92  (0.85-0.99) | 4,908 | 17 | 0.87  (0.75-0.99) | 4,464 | 21 | 0.87  (0.79-0.94) | 376 | 1 | 1.00  (*) | 312 | 0 | * | 290 | 1 | 1.00  (*) |
|  |  | M1 | 6,715 | 163 | 0.80  (0.76-0.84) | 4,908 | 119 | 0.79  (0.75-0.84) | 4,464 | 144 | 0.73  (0.69-0.78) | 376 | 12 | 0.76  (0.58-0.93) | 312 | 4 | 0.56  (0.18-0.94) | 290 | 9 | 0.88  (0.77-0.91) |
|  | South Asian | R2^+^ \| M1 | 1,647 | 49 | 0.85  (0.79-0.91) | 1,210 | 37 | 0.81  (0.73-0.89) | 924 | 30 | 0.67  (0.55-0.79) | 1,453 | 42 | 0.80  (0.71-0.88) | 1,256 | 33 | 0.64  (0.53-0.75) | 1,125 | 37 | 0.64  (0.52-0.75) |
|  |  | R2^+^ | 1,647 | 8 | 0.96  (0.91-1.00) | 1,210 | 3 | 0.90  (0.73-1.00) | 924 | 7 | 0.70  (0.56-0.84) | 1,453 | 1 | 0.73  (*) | 1,256 | 5 | 0.79  (0.62-0.97) | 1,125 | 4 | 0.93  (0.87-0.99) |
|  |  | M1 | 1,647 | 43 | 0.84  (0.77-0.91) | 1,210 | 37 | 0.81  (0.73-0.89) | 924 | 24 | 0.66  (0.51-0.80) | 1,453 | 41 | 0.80  (0.71-0.88) | 1,256 | 31 | 0.64  (0.52-0.76) | 1,125 | 36 | 0.63  (0.51-0.75) |
|  | Other Asian | R2^+^ \| M1 | 1,608 | 30 | 0.84  (0.74-0.93) | 1,182 | 15 | 0.82  (0.68-0.95) | 889 | 16 | 0.81  (0.70-0.93) | 125 | 3 | 0.89  (0.68-1.00) | 110 | 3 | 0.92  (0.83-1.00) | 104 | 3 | 0.82  (0.72-0.91) |
|  |  | R2^+^ | 1,608 | 5 | 1.00  (0.99-1.00) | 1,182 | 2 | 0.98  (0.97-1.00) | 889 | 6 | 0.90  (0.77-1.00) | 125 | 2 | 0.99  (0.97-1.00) | 110 | 2 | 0.96  (0.92-1.00) | 104 | 2 | 0.65  (0.55-0.74) |
|  |  | M1 | 1,608 | 27 | 0.82  (0.72-0.92) | 1,182 | 14 | 0.80  (0.66-0.95) | 889 | 12 | 0.78  (0.64-0.93) | 125 | 3 | 0.90  (0.72-1.00) | 110 | 3 | 0.92  (0.83-1.00) | 104 | 3 | 0.80  (0.69-0.91) |
|  | Mixed | R2^+^ \| M1 | 561 | 8 | 0.83  (0.66-0.99) | 400 | 12 | 0.83  (0.70-0.96) | 339 | 7 | 0.89  (0.76-1.00) | 78 | 4 | 0.97  (0.92-1.00) | 56 | 0 | * | 43 | 0 | * |
|  |  | R2^+^ | 561 | 2 | 0.97  (0.91-1.00) | 400 | 5 | 0.89  (0.74-1.00) | 339 | 5 | 0.84  (0.60-1.00) | 78 | 0 | * | 56 | 0 | * | 43 | 0 | * |
|  |  | M1 | 561 | 7 | 0.80  (0.62-0.98) | 400 | 10 | 0.84  (0.69-0.98) | 339 | 6 | 0.87  (0.74-1.00) | 78 | 4 | 0.96  (0.92-1.00) | 56 | 0 | * | 43 | 0 | * |
|  | Other | R2^+^ \| M1 | 586 | 6 | 0.85  (0.64-1.00) | 418 | 4 | 0.77  (0.35-1.00) | 328 | 8 | 0.68  (0.44-0.92) | 50 | 5 | 0.94  (0.87-1.00) | 34 | 3 | 1.00  (*) | 27 | 1 | 0.53  (*) |
|  |  | R2^+^ | 586 | 1 | 1.00  (*) | 418 | 0 | *  (*) | 328 | 1 | 0.19  (*) | 50 | 2 | 0.91  (0.82-0.99) | 34 | 2 | 1.00  (*) | 27 | 0 | * |
|  |  | M1 | 586 | 6 | 0.85  (0.74-1.00) | 418 | 4 | 0.77  (0.35-1.00) | 328 | 8 | 0.68  (0.44-0.92) | 50 | 3 | 0.96  (0.88-1.00) | 34 | 1 | 0.94  (*) | 27 | 1 | 0.54  (*) |
|  | Not Known | R2^+^ \| M1 | 415 | 5 | 0.68  (0.29-1.00) | 229 | 4 | 0.42  (0.18-0.65) | 215 | 6 | 0.96  (0.92-1.00) | 567 | 15 | 0.93  (0.88-0.98) | 448 | 8 | 0.70  (0.50-0.90) | 376 | 14 | 0.78  (0.65-0.91) |
|  |  | R2^+^ | 415 | 0 | * | 229 | 0 | * | 215 | 2 | 0.98  (0.94-1.00) | 567 | 4 | 0.92  (0.79-1.00) | 448 | 2 | 0.69  (0.10-1.00) | 376 | 4 | 1.00  (0.99-1.00) |
|  |  | M1 | 415 | 5 | 0.68  (0.30-1.00) | 229 | 4 | 0.41  (0.18-0.65) | 215 | 6 | 0.96  (0.93-0.99) | 567 | 12 | 0.93  (0.88-0.98) | 448 | 6 | 0.73  (0.56-0.91) | 376 | 12 | 0.73  (0.58-0.88) |

Outcomes with a **reduction** in the **AUROC ≥0.10** compared to the whole cohort are highlighted in **bold**. +ve=Positives. *Defining AUROC or confidence interval not possible. AUROC=Area-under-the receiver operating characteristic. CI=Confidence interval. R2^+^ | M1=Referable DR or maculopathy. R2^+^=Referable DR. M1=Referable maculopathy. Confidence intervals (95%) calculated using DeLong’s method.
