# Supplementary material for: Predicting 1, 2 and 3 year emergent referable diabetic retinopathy and maculopathy using deep learning
Source: Commun Med (Lond). 2024 Aug 21;4:167. doi: 10.1038/s43856-024-00590-z (PMC11339445; doi:10.1038/s43856-024-00590-z)
Supplement: Supplementary file 8 — Reporting summary [file 43856_2024_590_MOESM8_ESM.pdf]

nature portfolio

Corresponding author(s): Paul Nderitu

Last updated by author(s): Feb 29, 2024

Reporting Summary

Nature Portfolio wishes to improve the reproducibility of the work that we publish. This form provides structure for consistency and transparency in reporting. For further information on Nature Portfolio policies, see our Editorial Policies and the Editorial Policy Checklist.

Statistics

For all statistical analyses, confirm that the following items are present in the figure legend, table legend, main text, or Methods section.

n/a	Confirmed	
☐	☒	The exact sample size (*n*) for each experimental group/condition, given as a discrete number and unit of measurement
☐	☒	A statement on whether measurements were taken from distinct samples or whether the same sample was measured repeatedly
☐	☒	The statistical test(s) used AND whether they are one- or two-sided<br>*Only common tests should be described solely by name; describe more complex techniques in the Methods section.*
☐	☒	A description of all covariates tested
☐	☒	A description of any assumptions or corrections, such as tests of normality and adjustment for multiple comparisons
☐	☒	A full description of the statistical parameters including central tendency (e.g. means) or other basic estimates (e.g. regression coefficient) AND variation (e.g. standard deviation) or associated estimates of uncertainty (e.g. confidence intervals)
☐	☒	For null hypothesis testing, the test statistic (e.g. *F*, *t*, *r*) with confidence intervals, effect sizes, degrees of freedom and *P* value noted<br>*Give P values as exact values whenever suitable.*
☒	☐	For Bayesian analysis, information on the choice of priors and Markov chain Monte Carlo settings
☒	☐	For hierarchical and complex designs, identification of the appropriate level for tests and full reporting of outcomes
☐	☒	Estimates of effect sizes (e.g. Cohen's *d*, Pearson's *r*), indicating how they were calculated

Our web collection on statistics for biologists contains articles on many of the points above.

Software and code

Policy information about availability of computer code

Data collection	Not applicable
Data analysis	Analyses were performed between November 2022 and June 2023 using SPSS v27 (IBM) and Python v3.9. Deep learning models were developed using opencv v4.5.5, albumentations v1.0.3, timm v0.4.12, torch v1.10.1, torchmetrics v0.6.2, and pytorch-lightning v1.5.8 python libraries, with model attributions computed using captum v0.4.1. Other python libraries used for data analyses included numpy v1.21.5, pandas v1.3.5, scikit-learn v1.0.2, matplotlib v3.5.1, scipy v1.7.3 and statsmodels v0.13.2. A Windows 10 Pro workstation with a 32 core Intel Xenon CPU, 128GB of RAM and x2 24GB Quadra RTX P6000 GPUs was used for DLS development and internal testing. External testing was performed on a cloud-based trusted research environment with a GPU with 12GB of RAM.

For manuscripts utilizing custom algorithms or software that are central to the research but not yet described in published literature, software must be made available to editors and reviewers. We strongly encourage code deposition in a community repository (e.g. GitHub). See the Nature Portfolio guidelines for submitting code & software for further information.

Data

Policy information about availability of data

All manuscripts must include a data availability statement. This statement should provide the following information, where applicable:

- Accession codes, unique identifiers, or web links for publicly available datasets
- A description of any restrictions on data availability
- For clinical datasets or third party data, please ensure that the statement adheres to our policy

De-identified data used for development and internal testing are not publicly available at present due to the absence of authorisation for public data sharing. However, the data used for external validation can be requested from HDRUK (INSIGHT) with access/approval dependant on meeting data governance and application criteria.

Human research participants

Policy information about studies involving human research participants and Sex and Gender in Research.

Reporting on sex and gender	Reporting uses sex descriptor throughout in keeping with its clinical coding from the data source.
Population characteristics	All popualtion characateristics descirbed in detail in the main text and supplementray.
Recruitment	No recruitment as all retrospective data.
Ethics oversight	Ethics approval from the East Midlands Leicester South Research Ethics Committee (REC, 20/EM/0250) were obtained prior to study commencement. Licensed access for external data (INSIGHT, Health Data Research UK) was approved by the West of Scotland REC 4 (20/ES/0087).

Note that full information on the approval of the study protocol must also be provided in the manuscript.

Field-specific reporting

Please select the one below that is the best fit for your research. If you are not sure, read the appropriate sections before making your selection.

☒ Life sciences ☐ Behavioural & social sciences ☐ Ecological, evolutionary & environmental sciences

For a reference copy of the document with all sections, see nature.com/documents/nr-reporting-summary-flat.pdf

Life sciences study design

All studies must disclose on these points even when the disclosure is negative.

Sample size	No prior sample size calculation for this deep learning bases study but 162,339 development-set eyes used to train the model, 27,996, and 6,928 eyes used for internal and external model testing.
Data exclusions	All exclusion described in detail in the main manuscript.
Replication	N/A - However multiple model development strategies were tried with details for all hyper parameters used for model development provided in the supplementary and main text.
Randomization	N/A - Retrospective cohort design
Blinding	N/A - Retrospective cohort design

Reporting for specific materials, systems and methods

We require information from authors about some types of materials, experimental systems and methods used in many studies. Here, indicate whether each material, system or method listed is relevant to your study. If you are not sure if a list item applies to your research, read the appropriate section before selecting a response.

Materials & experimental systems

n/a	Involved in the study
☒	☐ Antibodies
☒	☐ Eukaryotic cell lines
☒	☐ Palaeontology and archaeology
☒	☐ Animals and other organisms
☒	☐ Clinical data
☒	☐ Dual use research of concern

Methods

n/a	Involved in the study
☒	☐ ChIP-seq
☒	☐ Flow cytometry
☒	☐ MRI-based neuroimaging
